# c-Jun Reprograms Schwann Cells of Injured Nerves to Generate a Repair Cell Essential for Regeneration

**DOI:** 10.1016/j.neuron.2012.06.021

**Published:** 2012-08-23

**Authors:** Peter J. Arthur-Farraj, Morwena Latouche, Daniel K. Wilton, Susanne Quintes, Elodie Chabrol, Ambily Banerjee, Ashwin Woodhoo, Billy Jenkins, Mary Rahman, Mark Turmaine, Grzegorz K. Wicher, Richard Mitter, Linda Greensmith, Axel Behrens, Gennadij Raivich, Rhona Mirsky, Kristján R. Jessen

**Affiliations:** 1 Department of Cell and Developmental Biology, University College London, Gower Street, London WC1E 6BT, UK; 2 Metabolomics Unit, CICbioGune, Parque Tecnológico de Bizcaia, 48160 Derio, Bizcaia, Spain; 3 Neuro-Oncology Group, Department of Immunology, Genetics and Pathology, Uppsala University, Dag Hammarskjölds väg 20, 751 85 Uppsala, Sweden; 4 Sobell Department of Motor Neuroscience & Movement Disorders, University College London Institute of Neurology, Queen Square House, London WC1N 3BG, UK; 5 Mammalian Genetics Laboratory, London Research Institute, CRUK, 44 Lincoln’s Inn Fields, London WC2A 3LY, UK; 6 Perinatal Brain Group, Department of Obstetrics and Gynaecology and Department of Cell and Developmental Biology, University College London, Gower Street, London WC1E 6BT, UK

## Abstract

The radical response of peripheral nerves to injury (Wallerian degeneration) is the cornerstone of nerve repair. We show that activation of the transcription factor c-Jun in Schwann cells is a global regulator of Wallerian degeneration. c-Jun governs major aspects of the injury response, determines the expression of trophic factors, adhesion molecules, the formation of regeneration tracks and myelin clearance and controls the distinctive regenerative potential of peripheral nerves. A key function of c-Jun is the activation of a repair program in Schwann cells and the creation of a cell specialized to support regeneration. We show that absence of c-Jun results in the formation of a dysfunctional repair cell, striking failure of functional recovery, and neuronal death. We conclude that a single glial transcription factor is essential for restoration of damaged nerves, acting to control the transdifferentiation of myelin and Remak Schwann cells to dedicated repair cells in damaged tissue.

## Highlights

► Schwann cell c-Jun is a master regulator of the PNS injury response ► c-Jun activates a defined repair program in Schwann cells of damaged nerves ► c-Jun controls transdifferentiation of differentiated Schwann cells to repair cells ► Schwann cell c-Jun is essential for neuronal survival and functional recovery

## Teaser

Unlike the central nervous system, injured peripheral nerves regenerate to restore function after injury. Arthur-Farraj et al. show that this repair potential depends on glial (Schwann) cell expression of the transcription factor c-Jun.

## Introduction

How transcription factors control cellular plasticity and maintain differentiation is currently of great interest, inspired by the success of experimental reprogramming, where remarkable phenotypic transitions can be induced by enforced expression of fate determining factors (Zhou and Melton, 2008). These findings raise a key question: to what extent are natural transitions in the state of differentiated cells also governed by specific transcription factors? Such phenotypic transitions are seen in tumorigenesis, dedifferentiation and transdifferentiation. They are also fundamental to tissue repair and regeneration, and in regenerative systems, a major focus of work is identification of gene programs that are selectively activated after injury and which impact the repair process.

The striking regenerative capacity of the PNS rests on the surprising plasticity of Schwann cells, and the ability of these cells to switch between differentiation states, a feature that is highly unusual in mammals (Jessen and Mirsky, 2005, 2008; Jopling et al., 2011).

In a process reminiscent of the radical injury responses of zebrafish cardiomyocytes or pigment cells of the newt iris, nerve injury, and loss of axonal contact causes mammalian Schwann cells to lose their differentiated morphology, downregulate myelin genes, upregulate markers of immature Schwann cells, and re-enter the cell cycle. This radical process of natural dedifferentiation has few if any parallels in mammalian systems.

At the same time as Schwann cells dedifferentiate, they upregulate genes implicated in promoting axon growth, neuronal survival, and macrophage invasion, and activate mechanisms to break down their myelin sheaths and transform morphologically into cells with long, parallel processes. This allows them to form uninterrupted regeneration tracks (Bands of Bungner) that guide axons back to their targets (Chen et al., 2007; Vargas and Barres, 2007; Gordon et al., 2009). Collectively, these events together with the axonal death that triggers them are called Wallerian degeneration. This response transforms the normally growth-hostile environment of intact nerves to a growth supportive terrain, and endows the PNS with its remarkable and characteristic regenerative potential. To complete the repair process, Schwann cells envelop the regenerated axons and transform again to generate myelin and nonmyelinating (Remak) cells.

Little is known about the transcriptional control of changes in adult differentiation states, including natural dedifferentiation and transdifferentiation, in any system (Jopling et al., 2011). In line with this, although Wallerian degeneration including the Schwann cell injury response are key to repair, the molecular mechanisms that control these processes are not understood (Chen et al., 2007; Jessen and Mirsky, 2008). Conceptually also, the nature of the Schwann cell injury response has remained uncertain, since the generation of the denervated Schwann cell is commonly referred to either as dedifferentiation or as activation. These terms highlight two distinct aspects of the process, namely loss of the differentiated Schwann cell phenotypes of normal nerves and gain of the regeneration promoting phenotype, respectively, without providing a framework for analysis and comparison with other regenerative models.

Here, we use mice with selective inactivation of the transcription factor c-Jun in Schwann cells to show that c-Jun is a global regulator of the Schwann cell injury response that specifies the characteristic gene expression, structure, and function of the denervated Schwann cell, a cell that is essential for nerve repair. Consequently, axonal regeneration and functional repair are strikingly compromised or absent when Schwann cell c-Jun is inactivated. Notably, the effects of c-Jun are injury specific, since c-Jun inactivation has no significant effects on nerve development or adult nerve function.

These observations provide a molecular basis for understanding Schwann cell plasticity, show that c-Jun is a key regulator of Wallerian degeneration, and offer conclusive support for the notion that glial cells control repair in the PNS. They also show that the Schwann cell injury response has much in common with transdifferentiation, since it represents the generation, by dedicated transcriptional controls, of a distinct Schwann cell repair phenotype, specialized for supporting axon growth and neuronal survival in injured nerves. Because these cells form the regeneration tracks called Bungner’s bands, we will refer to them as Bungner cells.

## Results

### c-Jun Controls the Molecular Phenotype of the Denervated Schwann Cell

**Figure 1:**
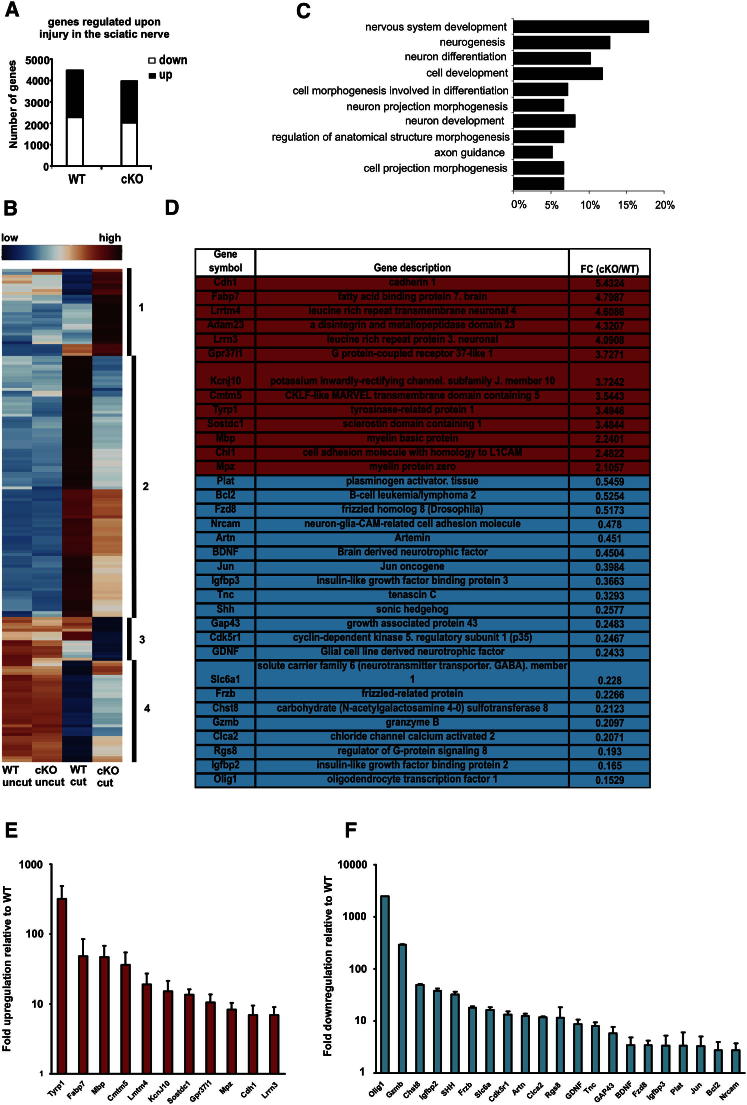
c-Jun Controls the Molecular Reprogramming of Schwann Cells in Injured Nerves (A) The number of genes in wild-type (WT) and mutant (cKO) nerves that show significant change in expression levels 7 days post-nerve cut, determined by microarray. The number of up- and downregulated genes is also indicated. Only genes that showed ≥1.5-fold change compared to uninjured nerves were considered. The gene screen data are an average of two independent experiments, each involving nerves pooled from 7–8 animals. (B) Heatmap showing expression of the 172 genes differentially regulated (≥1.5-fold threshold; microarray) in the distal stump of cut WT and c-Jun mutant mice. Comparison of cut WT and cut mutant nerves shows four main types of disregulation in c-Jun mutants (categories 1–4 indicated on the heatmap): (1) enhanced activation, (2) failure of activation, (3) enhanced downregulation, (4) failure of downregulation. (C) Enriched gene ontology (GO) categories for the 172 differentially regulated genes. (D) Subset of the 172 differentially regulated genes chosen for further analysis. This includes the 10 most upregulated and 10 most downregulated genes comparing the distal stump of WT and mutants, and 14 other genes chosen for potential relevance in nerve injury. Genes expressed at higher levels in the distal stump of mutants versus WT are highlighted in red. Genes expressed at lower levels in mutants versus WT are highlighted in blue. FC (cKO/WT) indicates fold change in expression levels in mutant versus WT distal stumps determined by microarray. (E) Genes overexpressed in the mutant: RT-QPCR determination of genes from (D) (red upper panel) showing fold increase in expression in the distal stump of mutants versus WT. Error bars: ± SEM; n = 3–6 pools of 3 animals/pool. (F) Genes underexpressed in mutants: RT-QPCR determination of genes from (D) (blue lower panel) showing fold decrease in expression in the distal stumps of mutants versus WT. Error bars: ± SEM; n = 3–6 pools of 3 animals/pool. See also Figures S1–S4 and Tables S1, S2, S3, and S5.

Earlier, we found that neonatal mice with conditional deletion of c-Jun in Schwann cells (c-Jun mutant mice) show delayed loss of myelin proteins and mRNA after nerve injury (Parkinson et al., 2008). This suggested that Schwann cell c-Jun might play an important role in specifying the phenotype of denervated Schwann cells. To test this comprehensively, we used Affymetrix whole-genome microarray to examine gene expression in the sciatic nerve of adult c-Jun mutant mice and control (WT) littermates and compared this with gene expression in denervated cells in the distal stump of transected nerves without regenerating axons, to avoid the complicating effects of axon-induced redifferentiation (Figure 1). We chose 7 days after injury since in regenerating mouse nerves this is near the mid-point of active axonal regrowth. Seven day denervated cells therefore represent the terrain that confronts regenerating axons in WT and mutant nerves.

Before injury, the nerves of adult c-Jun mutant mice were normal on the basis of a number of criteria. Thus, the numbers of myelinated and unmyelinated axons (see Figures 4E and 4F), myelinating Schwann cells and Remak bundles (see Table S1 available online), g-ratios (Figure S1), sciatic functional index (SFI) (see Figure 7E), motor performance in a rotarod test (unpublished), and responses to heat and light touch (see Figures 7B and 7C) were similar to WT controls. While c-Jun was excised from almost all Schwann cells (Parkinson et al., 2008), c-Jun expression in neurons, macrophages, and fibroblasts was normal, and the rate of axonal disintegration after cut was similar in WT and mutants (Figures S2 and S3).

The close similarity between WT and mutant nerves was confirmed by the Affymetrix screen (Figure 1), since only two genes (keratin 8 and desmoplakin) were differentially expressed. Furthermore, following injury, a comparable number of genes changed expression in WT and c-Jun mutants (Figure 1A). Importantly, however, comparison of the distal stumps of WT and c-Jun mutants revealed 172 significant differences in gene expression (Figure 1 and Tables S2 and S3). The differentially regulated genes included genes which have been implicated in regeneration and trophic support such as *BDNF*, *GDNF*, *Artn*, *Shh*, and *GAP-43* that failed to upregulate after injury, together with genes that failed to downregulate normally after injury such as the myelin genes *Mpz*, *Mbp*, and *Cdh1* (also known as *E-cadherin*). Gene ontology analysis indicated that known functions of these 172 genes were particularly related to neuronal growth and regeneration (Figure 1C).

**Figure 2:**
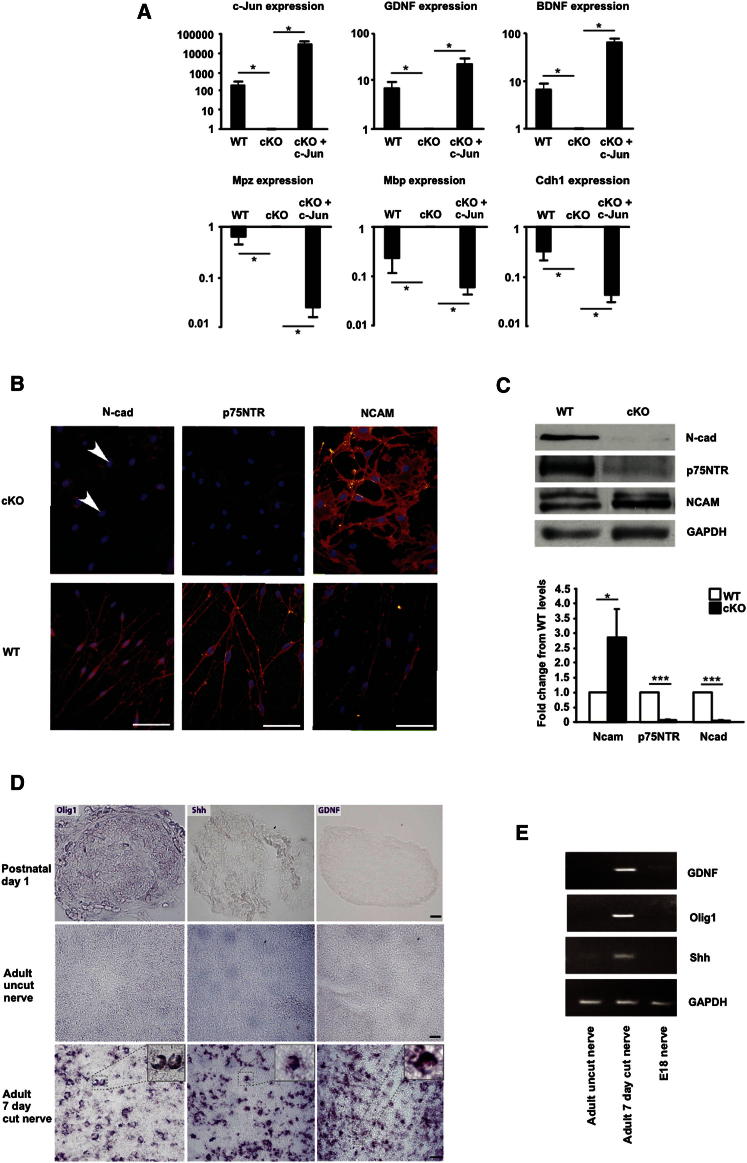
c-Jun-Dependent Regulation of Schwann Cell Genes and Proteins (A) In purified Schwann cell cultures, c-Jun suppresses myelin genes but activates genes of denervated cells. Graphs show RT-QPCR measurements of six differentially regulated genes in cells from control mice with physiological levels of c-Jun (WT), in cells from mutants lacking c-Jun (cKO), and in mutant cells infected with c-Jun adenovirus to re-express c-Jun (cKO + c-Jun). The y axis shows fold difference in expression levels. Error bars: ± SEM; ^∗^p < 0.05, n = 4. (B and C) Posttranscriptional control of protein expression by c-Jun. (B) Immunolabeling of Schwann cell cultures (5 days in vitro) from p8 WT and mutant nerves. Three proteins characteristically expressed by denervated Schwann cells are shown. Note suppression of N-cadherin (N-cad) and p75NTR but overexpression of NCAM in mutant cells. (Bar: 50 μm). (C) Western blots of distal stump nerve extracts from WT and mutants (cKO) 7 days after cut show a similar expression pattern. Lower panel: quantitation. Error bars: ± SEM; ^∗^p < 0.05; ^∗∗∗^p < 0.001, n = 3. (D and E) Immature and denervated cells differ in gene expression. (D) In situ hybridization using probes against *Olig1* (left panel), *Shh* (middle panel), and *GDNF* (right panel). mRNAs of these are virtually absent from uninjured newborn and adult sciatic nerve but highly upregulated in distal stumps of adult nerve 3 days after transection. Numerous positive cells display the half moon shape typical of Schwann cells (insets in bottom panels). (Bar: 25μm). (E) Agarose gel electrophoresis of *GDNF*, *Olig1*, *Shh*, and *GAPDH* products amplified by RT-QPCR from cDNA extracted from WT sciatic nerves of E18 and adult mice and from distal stumps of adult mice 7 days postcut. See also Tables S4 and S5.

We selected 32 of the 172 disregulated genes for further analysis by RT-QPCR. In every case this confirmed the disregulation shown by the microarray data (Figures 1D–1F and Table S3). Six of the thirty-two genes were then analyzed in purified Schwann cell cultures. Comparison of c-Jun mutant and WT cells confirmed the regulation seen in the distal stumps. Furthermore, as predicted, enforced c-Jun expression in c-Jun mutant cells, by adenoviral gene transfer, activated *c-Jun*, *BDNF*, and *GDNF* expression but suppressed *Chd1*, *Mpz*, and *Mbp* expression (Figure 2A). These results show directly that c-Jun regulates these genes in Schwann cells, demonstrates that this control is independent of the nerve environment, and confirms results obtained by microarray and RT-QPCR.

Lastly, we found that three proteins implicated in regeneration, N-cadherin, p75NTR, and NCAM, were disregulated in cut mutant nerves, although their mRNAs were normally expressed. Injured mutant nerves expressed strongly reduced N-cadherin and p75NTR but elevated levels of NCAM (Figures 2B and 2C). Sox2 protein, which, like c-Jun, is upregulated in WT Schwann cells of injured nerves (Parkinson et al., 2008), remained normally upregulated in injured nerves of c-Jun mutants (Figure S4).

Denervated Schwann cells in injured adult nerves are often considered similar to immature Schwann cells in developing nerves. However, the immature cells for instance do not share the axon guidance, myelin breakdown and macrophage recruitment functions of denervated cells, and these cells differ in molecular expression (Jessen and Mirsky, 2008). To explore the idea that the denervated cell represents a distinct Schwann cell phenotype regulated by c-Jun, we examined three genes, *Olig1*, *Shh*, and *GDNF*, which showed strong, c-Jun-dependent activation in denervated cells (Figure 1D). Using RT-QPCR and in situ hybririsization we confirmed strong expression of these genes in WT adult denervated cells, but found that they were not (*Olig1* and *Shh*) or borderline (*GDNF*) detectable in immature Schwann cells (from WT embryo day 18 nerve). They were also essentially absent from uncut nerves (Figures 2D and 2E and Table S4). This supports the notion that denervated adult Schwann cells and immature Schwann cells in perinatal nerves represent distinct cell types. It shows also that c-Jun takes part in controlling the distinctive molecular profile of the adult denervated cell. The response of neonatal cells to injury remains to be determined.

Together these results show that c-Jun controls the molecular reprogramming that transforms mature Schwann cells to the denervated cell phenotype following injury. This includes the regulation of genes that differentiate denervated from immature cells and extends to the posttranscriptional control of protein expression.

### c-Jun Controls the Structure of Regeneration Tracks (Bands of Bungner)

**Figure 3:**
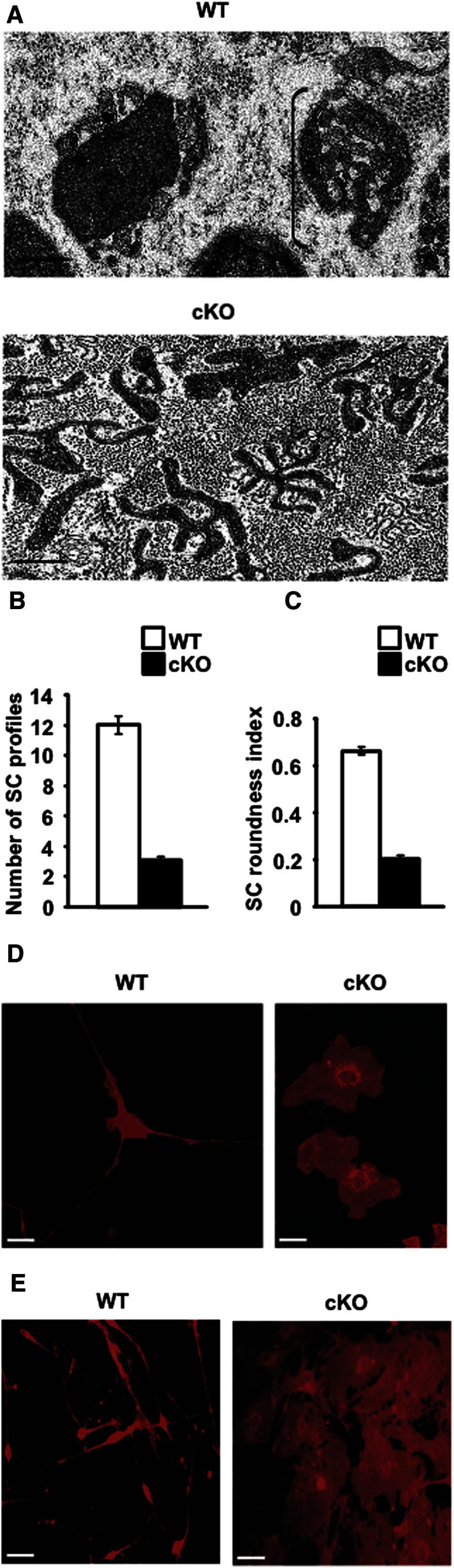
c-Jun Controls the Structure of Denervated Schwann Cells In Vivo and in Culture (A) Electron micrographs of distal stumps of WT and c-Jun mutant (cKO) sciatic nerve 28 days after transection (no regeneration). The WT nerve contains classic regeneration tracks (Bands of Bungner; an example is bracketed, showing several Schwann cell processes within a basal lamina tube). These do not form in the mutant, which instead contains irregular and flattened cellular profiles. Bar: 1 μm. (B) The number of cellular profiles per regeneration track is sharply reduced in the mutant. Error bars: ± SEM; p < 0.05, n = 4. (C) Mutant cellular profiles are flatter (lower roundness index). Error bars: ± SEM; p < 0.05, n = 4. (D and E) In vitro, WT Schwann cells from neonatal nerves show typical bi- or tripolar morphology, but mutant cells (cKO) are flat, irrespective of whether they are taken from c-Jun mutants (D) or obtained by infecting c-Jun^f/f^ cells with CRE-adenovirus (E). Cells are labeled with β1 integrin antibodies. Bar: 20 μm.

Denervated Schwann cells form cellular columns that replace the axon-Schwann cell units of intact nerves and serve as substrate for growing axons. We examined these structures by electron microscopy in the distal stump 4 weeks after cut. Because these cells have been without axonal contact for 4 weeks they are comparable to the cells encountered by growing axons in distal parts of crushed nerves in the c-Jun mutant where regeneration is delayed beyond the normal 3–4 week period, while at this time WT nerves have just reached their targets. We found that the structure of these regeneration tracks is strikingly abnormal in c-Jun mutants (Figure 3A). There are many fewer cell profiles per column, indicating reduced process formation (Figure 3B) and the cells have flattened as confirmed by reduction in the roundness index of cell profiles in vivo (Figure 3C). Flattening and paucity of processes are also seen even in *c-Jun*^*−/−*^ cells from neonatal nerves in vitro (Figures 3D and 3E). Therefore, this is a robust phenotype that does not depend on long term denervation in vivo.

Thus, c-Jun is an cell-intrinsic determinant of Schwann cell morphology that controls the structure of the essential regeneration tracks that guide growing axons back to correct targets.

### In c-Jun Mutants, Injury Results in Extensive Death of Sensory (DRG) Neurons

c-Jun specification of gene expression and morphology of denervated cells suggested that Schwann cell c-Jun might exert a decisive control over nerve repair. Because survival of injured neurons is the basis for repair, we measured the survival of small and large dorsal root sensory (DRG) neurons following sciatic nerve crush at the sciatic notch. We counted axons in L4 dorsal roots (Coggeshall et al., 1997) and the tibial nerve, and neuronal somas and nucleoli in DRGs. Comparable results were obtained using all methods.

**Figure 4:**
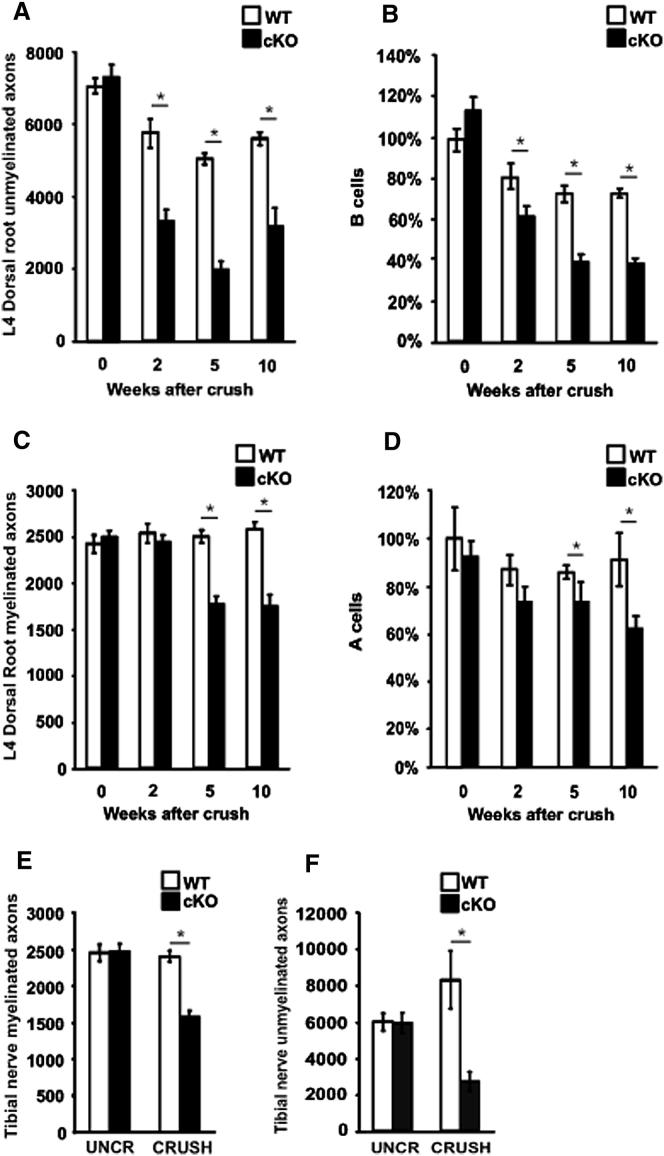
Axonal Injury Results in Extensive Neuron Death in c-Jun Mutants (A and B) Number of unmyelinated axons in L4 dorsal roots (A) and number of small neuronal cell body profiles (B cells) in corresponding DRGs (B), expressed relative to number of B cells in uninjured WT mice. The data show before (0) and at different times after injury. Error bars: ± SEM; ^∗^p < 0.05, n = 4). (C and D) Number of myelinated axons in L4 dorsal roots (C) and large neuronal cell body profiles (A cells) in corresponding DRGs (D), expressed relative to the number of A cells in uninjured WT mice. The data show before (0) and at different times after injury. Error bars: ± SEM; ^∗^p < 0.05, n = 4. (E and F) Number of myelinated (E) and unmyelinated (F) axons in tibial nerves (midthigh level) before injury and 10 weeks postcrush in WT and mutant mice. Error bars: ± SEM; ^∗^p < 0.05, n = 4. Note that axon and neuron numbers are normal in c-Jun mutants before injury, but significantly reduced post nerve crush. See also Figure S2.

Axon counts in WT dorsal roots showed that 20%–25% of the unmyelinated axons were lost following crush, as expected (Coggeshall et al., 1997). In contrast, 55%–60% of these axons were lost in c-Jun mutants, showing increased death of small DRG neurons in the mutant. This was confirmed by corrected (Abercrombie, 1946) counts of B neuron profiles in DRGs, showing 25%–30% loss in WT but 45%–65% loss in the mutants (Figures 4A and 4B).

The number of myelinated axons in dorsal roots remained unchanged in injured WT mice as expected (Coggeshall et al., 1997). But surprisingly, in the mutants the number of myelinated axons was reduced by 30%–35%, indicating death of large DRG cells. In confirmation, the corrected number of large A cell profiles in DRGs was reduced by about 40% in the mutants. The number of these profiles did not change significantly in injured WT (Figures 4C and 4D). We also carried out counts on DRG sections using nucleoli as the counted entity, an approach that theoretically provides increased accuracy. Nucleoli in A type DRG neurons from uncut WT (n = 3), 10 week cut WT (n = 3), and 10 week cut c-Jun mutant (n = 3) mice were counted, corrected (Abercrombie, 1946), and expressed as percentage of uncut WT. This showed a 12% reduction in cut WT, (not significant; p > 0.40) but a 50% reduction in the c-Jun mutant (highly significant; p < 0.017). This provides a third line of evidence (in addition to counts of myelinated axons in dorsal roots and cell profiles) for the notion that nerve injury results in the loss of A type DRG neurons in mice that selectively lack c-Jun in Schwann cells.

Consistent with neuronal death, the number of myelinated axons in the mutant tibial nerve 10 weeks after crush was reduced by about 35% and unmyelinated axons were reduced by about 65%, both compared to crushed WT controls (Figures 4E and 4F).

These experiments show that without Schwann cell c-Jun, small, unmyelinated DRG neurons are about twice as likely to die following axonal damage. Significantly, about a third of the large, myelinated DRG neurons also die in crushed c-Jun mutants, although none die in injured WT controls, and in other studies these cells are resistant to death following axonal damage (Tandrup et al., 2000). These experiments establish that a key function of denervated Schwann cells is to prevent the death of injured neurons and that this rescue depends on c-Jun activation.

### Failure of Axon Growth and Target Reinnervation in c-Jun Mutants

**Figure 5:**
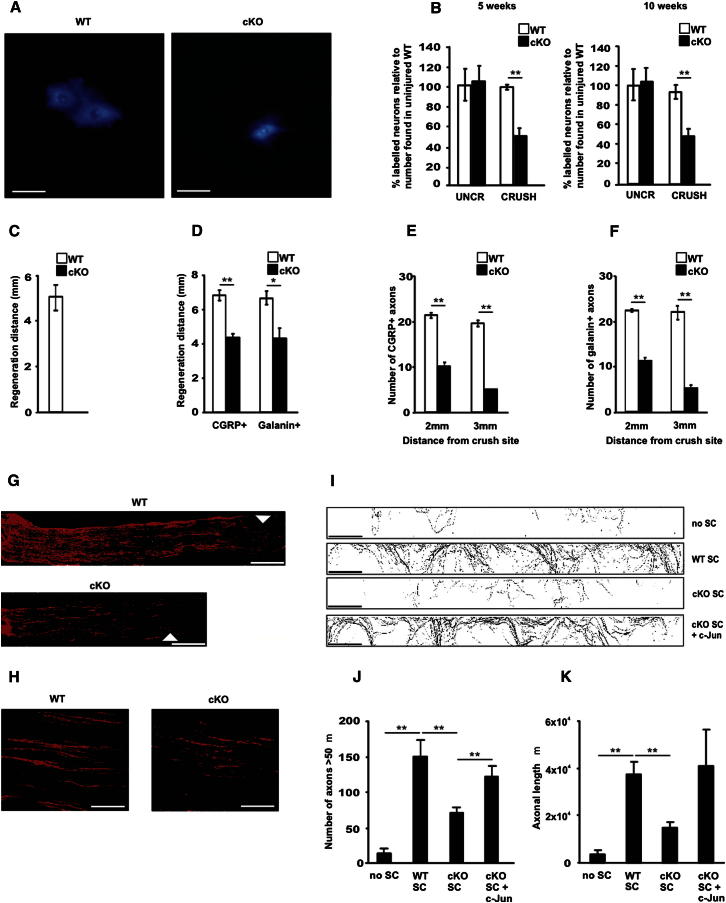
Axonal Regeneration Failure in c-Jun Mutants (A) A reduced number of backfilled motoneurons is seen in the ventral horn of c-Jun mutants after True Blue injection into the tibialis anterior muscle 10 weeks post-nerve crush. Bar: 100 μm. (B) Quantification of backfilled neurons 5 and 10 weeks after crush. Abercrombie correction applied. Note reduced number of labeled neurons in the L2–L6 region of the spinal cord. Error bars: ± SEM; ^∗∗^p < 0.01, n = 4. (C) Regeneration failure in mutants judged by nerve pinch test 4 days post sciatic nerve crush. Error bar: ± SEM; n = 4. (D–H) CGRP^+^ or galanin^+^ regenerating axon numbers are reduced in mutants 4 days postcrush. Micrograph (G) shows CGRP labeling of nerve fronts (arrows; bar: 1 mm) in WT and mutant nerves; (H) shows axons 3 mm from the crush site (bar: 100 μm). Regeneration delay is quantified by measuring the distance from crush traveled by the longest axon (D), or by counting how many axons extend 2 or 3 mm from the crush site (E and F). For (D) ^∗^p < 0.05; ^∗∗^p < 0.01, n = 4; for (E) and (F) ^∗∗^p < 0.01, n = 4. (I) In microfluidic chambers, WT Schwann cells and mutant cells with enforced c-Jun expression promote axon growth relative to no cells or mutant cells. Each trace shows axon growth into a side chamber from a central compartment containing neuronal cell bodies. The four types of side chamber are as follows: (no SC) chamber with no Schwann cells; (WT SC) chamber with WT Schwann cells with normal constitutive c-Jun expression; (cKO SC) chamber with cells from c-Jun mutants (no c-Jun), and (cKO SC + c-Jun) chamber with mutant cells infected with c-Jun adenovirus to re-express c-Jun. Bar: 250 μm. (J) Quantification of the number of axons longer than 50 μm growing into the side compartment in all conditions shown in (I). (K) Quantification of the total area covered by axons in the side compartment in all conditions depicted in (I). Error bars: ± SEM; ^∗∗^p < 0.01, n = 3. See also Figure S2.

The number of myelinated axons in ventral roots of both WT and mutant mice remained unchanged following sciatic nerve crush (Table S6). Therefore, unlike DRG neurons, survival of injured ventral horn motoneurons is independent of Schwann cell c-Jun. Nevertheless, the corrected (Abercrombie, 1946) counts of motoneurons that reconnected with the target muscle showed a large reduction in the mutant, even as late as 10 weeks after injury, reaching only about 55% of that in controls, judged by motoneuron backfilling (Figures 5A and 5B). This indicates that in the mutants, axonal regeneration by surviving neurons is severely and permanently compromised.

To analyze regeneration, we examined sciatic nerves 4 days after crush, using the nerve pinch test and by quantifying the number and length of axons in longitudinal sections immunolabeled by CGRP or galanin antibodies to label regenerating DRG and motoneurons. This showed a strong decrease in axon outgrowth in the mutants compared to WT (Figures 5C–5H).

Regeneration in the mouse sciatic nerve is independent of Schwann cell proliferation (Kim et al., 2000; Yang et al., 2008). Nevertheless, because c-Jun contributes to proliferation in vitro (Parkinson et al., 2008), we counted Schwann cells in WT and mutant distal stumps (Table S7). In crushed, actively regenerating nerves (14 days after injury) Schwann cell numbers were not significantly different between WT and mutants; both were elevated 5- to 6-fold compared to uncut nerves. Four days after crush, cell numbers were higher in WT nerves, while 7 days after cut, again the difference between mutants and WT was not significant. The tendency toward lower Schwann cell numbers in the mutants is in line with the involvement of c-Jun in proliferation (Parkinson et al., 2004).

Together this shows that in the absence of Schwann cell c-Jun, the regeneration of axons from surviving neurons is severely reduced, leading to a permanent deficit in the number of neurons that reconnect with denervated targets. The observation that that Schwann cell numbers in regenerating mutant nerves are elevated up to 5-fold compared to uninjured nerves, together with the independence of regeneration from elevated Schwann cell numbers (Kim et al., 2000; Yang et al., 2008) and analysis using microfluidic chambers (following section), shows that that regeneration failure in c-Jun mutants is not caused by lack of Schwann cells.

### c-Jun Controls Direct Interactions between Axons and Schwann Cells

A nerve is a complex cell community. We therefore used microfluidic chambers containing neurons and purified Schwann cells to test whether the poor axon growth in mutants was caused by disturbance of direct axon-Schwann cell interactions or whether the effect depended on other cells.

Axon regeneration by axotomized, adult WT DRG neurons was strongly stimulated by control Schwann cells relative to laminin substrate alone, as expected (Figures 5I–5K). The c-Jun mutant cells, however, were ineffective, the number and area of axons extending on their surface falling to only 40%–50% of that seen on WT cells. Importantly, reactivation of c-Jun in mutant cells by adenoviral gene transfer, fully restored axon number and length to WT levels.

These experiments show that injury-activated Schwann cell c-Jun controls direct communication between Schwann cells and growing neurites.

### Schwann Cell c-Jun Controls Myelin Clearance

We have shown that c-Jun controls three important functions of denervated Schwann cells, formation of regeneration tracks, support of neuronal survival, and promotion of axon regrowth. A fourth major role classically ascribed to these cells is removal of myelin and associated growth inhibitors, a task they accomplish by breaking down myelin early after injury and indirectly by instructing macrophages to complete myelin clearance (Hirata and Kawabuchi, 2002).

**Figure 6:**
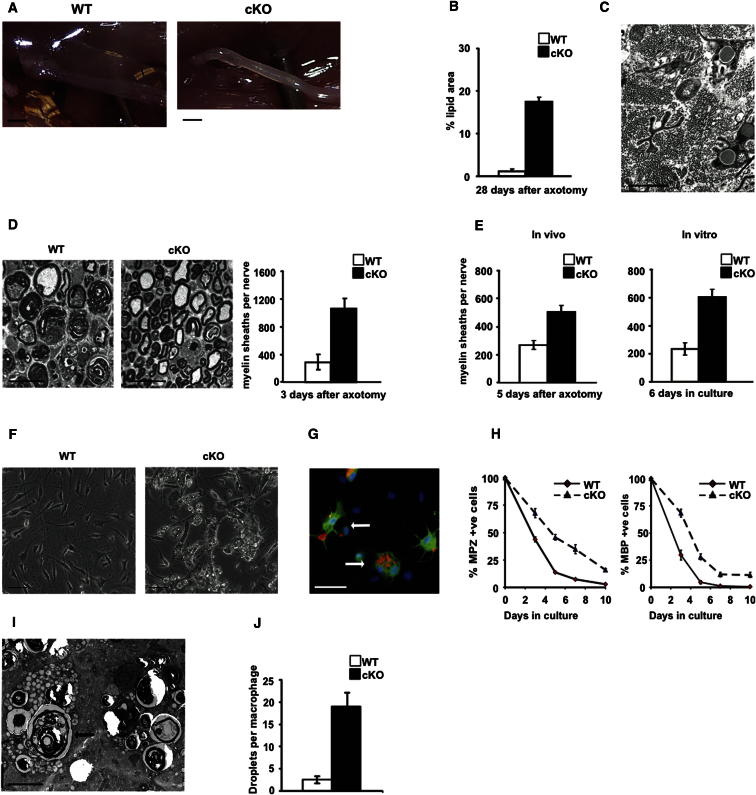
Myelin Clearance Is Slow in Injured c-Jun Mutant Nerves (A) Unlike WT nerves, mutant nerves (cKO) are not translucent 4 weeks postcut, indicating lipid retention. Bar: 2 mm. (B) Lipid persistence in mutant nerves shown by quantification of osmium stained area in transverse sections of sciatic nerve 4 weeks post cut (no regeneration). Error bars: ± SEM; p < 0.001, n = 4. (C) Electron micrograph showing lipid droplets in denervated Schwann cells of mutant nerves 4 weeks post cut. Bar: 2 μm. (D) Electron micrograph showing relative preservation of intact myelin sheaths in mutants 3 days postcut, 3 mm from cut site. Right, counts of intact sheaths in tibial nerves of WT and mutants 3 days after cut. Error bars: ± SEM; p < 0.05, n = 5. Bar: 20 μm. (E) Myelin sheath counts in tibial nerves following axotomy in vivo and in nerve segments in vitro, as indicated. Note that sheath preservation in the mutant does not depend on blood-born macrophages. Error bars: ± SEM; p < 0.05, n = 4. (F) Normal myelin breakdown fails in mutant Schwann cells, shown by persistence of myelin debris in mutant cells in phase-contrast micrographs of cultures from p8 WT and mutant nerves and maintained 6 days in vitro. Bar: 50 μm. (G) Immunolabeling of mutant Schwann cells (green; S100 antibodies), bloated with myelin debris (red; MBP antibodies) (arrows indicate two cells). Bar: 50 μm. (H) Counts of cells containing the myelin proteins MPZ and MBP at different times after plating cells from WT and mutant p8 nerves show delay in myelin protein clearance by mutant Schwann cells. 0 = 3 hr after plating. Differences between cKO and WT were significant at all time points. Error bars: ±SEM; p < 0.001, (two way ANOVA), n = 5. (I) Electron micrograph shows the persistence in mutant nerves of large, foamy macrophages (example arrowed). Bar: 5 μm. (J) Lipid droplet counts in macrophages in tibial nerve sections 28 days postcut (no regeneration), show a strong delay in lipid clearance by macrophages in the mutant. Error bars: ±SEM; p < 0.05, n = 4. See also Figures S3 and S5.

We found that myelin clearance was substantially delayed in mutants. Four weeks after sciatic nerve transection (without regeneration), the distal stump of WT nerves was translucent, while mutant nerves remained gray/white (Figure 6A). Osmium stained lipid debris occupied about 10-fold larger area in the mutant than WT nerves (Figure 6B).

Electron microscopy revealed that although transected mutant nerves did not contain intact myelin, many Schwann cells contained lipid droplets, a late product of myelin breakdown (Figure 6C). This was not seen in 4 week transected WT controls. We therefore tested whether myelin breakdown was impaired in mutant Schwann cells. First, in cut adult nerves, the loss of myelin sheaths was delayed in the mutants (Figure 6D). This was not due to infiltrating macrophages, because the difference between WT and mutants was fully maintained when the cut nerves were maintained in vitro (Figure 6E). Second, this delay was confirmed by slower breakdown of myelin basic protein (MBP) in vivo (Figures S5A and S5B). Third, when myelinating cells from postnatal day 8 nerves were cultured, myelin proteins were broken down slowly by c-Jun mutant cells compared to WT, and mutant cultures contained many Schwann cells bloated with myelin debris (Figures 6F–6H). Both types of culture contained similar numbers of F4/80^+^ macrophages (5.6+/−1.8% and 5.8+/−1.7%, n = 4, in WT and mutants, respectively, at 3 days in vitro), and no F4/80^+^ cells containing myelin proteins were seen, suggesting macrophages are not significantly involved in myelin breakdown in these experiments.

These findings show that c-Jun mutant Schwann cells are deficient in their ability to break down myelin.

Surprisingly, abnormalities in myelin breakdown extended to the macrophage compartment, although the macrophages are genetically normal (Figure S2). Four weeks after cut, macrophages in the mutants contained large amounts of myelin debris, and counts of lipid droplets per macrophage showed that they were about 7 times more numerous than in WT (Figures 6I and 6J). Furthermore, the number of very large (>150 μm^2^) foamy macrophages bloated with debris was elevated 3-fold in mutant nerves at this time point.

Macrophage numbers in the distal stump were strongly elevated in both WT and c-Jun mutant nerves after injury. Three days after cut, their number close to the injury site was significantly higher in WT mice, and a migration assay using Boyden chambers showed that WT nerves attracted more macrophages than mutant nerves (Figures S5C and S5D). But at 1 and 6 weeks after cut, the number of macrophages was similar in WT and mutants, and at 4 and 14 days after crush, macrophage numbers were not significantly different (Table S7). RT-QPCR of cytokines in the distal stumps 36 hr after cut did not reveal significant differences between WT and mutants (Figure S5E). This indicates that c-Jun mutants do not suffer from a major failure in macrophage recruitment. Reduced numbers shortly after injury in the mutant might relate to lower Schwann cell numbers rather than to significant disturbance in the expression of macrophage attractants by individual cells.

These results show that injured c-Jun mutant nerves develop substantial problems with myelin clearance. This is evident not only in Schwann cells but also in macrophages, an observation that suggests a role for Schwann cells in the control of macrophage activation and myelin degradation.

### Functional Recovery Fails in the Absence of c-Jun in Schwann Cells

Previous sections show that injury-activated Schwann cell c-Jun controls the generation of the denervated Schwann cell, and controls key cellular interactions during Wallerian degeneration and nerve repair. The end result of this process is functional recovery. This is remarkably effective in rodents, where full recovery is seen 3–4 weeks after crush. We found that this essential feature of peripheral nerves was abolished or strikingly compromised in c-Jun mutant mice.

To measure sensory function, we used the following: (1) toe pinching (pressure), (2) Von Frey hairs (light touch), and (3) the Hargreaves test (temperature).

**Figure 7:**
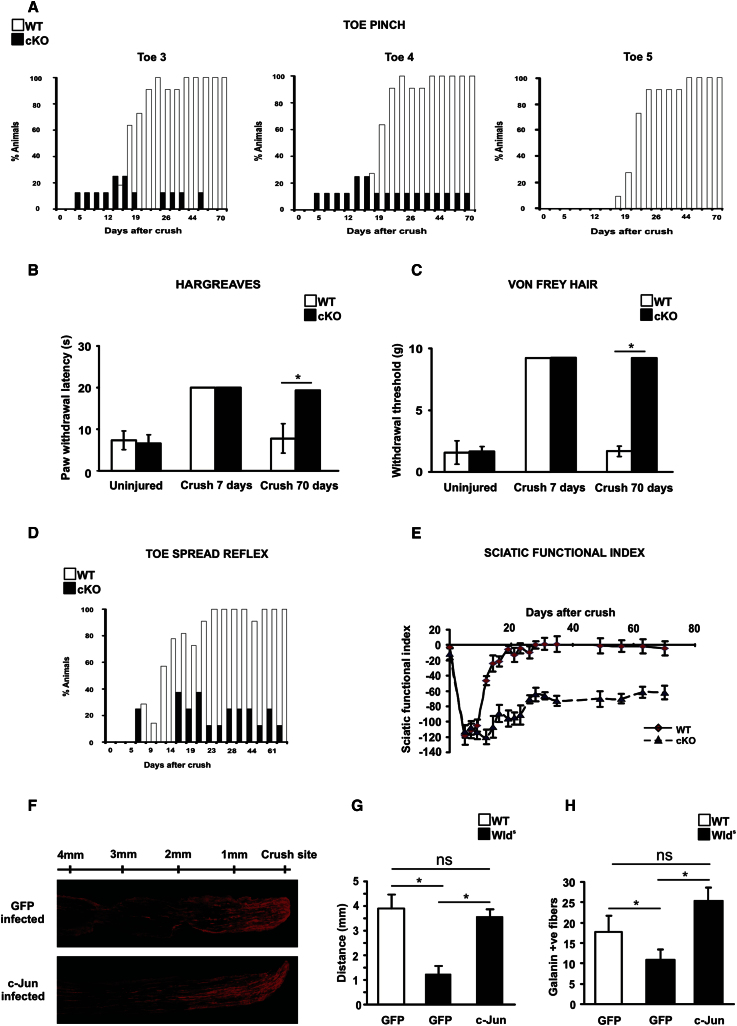
Functional Recovery Fails in c-Jun Mutants (A) Percentage of mice responding to pinching of distal parts of toes 3, 4, and 5 after nerve crush. The difference between WT and mutant (cKO) mice was significant from day 21 to day 70, p < 0.01 (two-way ANOVA), n = 5. (B and C) Sensitivity to heat (B), and light touch (C) quantified in mice before and after nerve crush. In animals assayed 7 days after crush, the assay was terminated at 20 s (Hargreaves test) and limited to the use of a hair weight of 8 g (Von Frey test). Mutants show normal sensation when uninjured, but no recovery after injury. Error bars: ±SEM; ^∗^p < 0.05, n = 4. (D) Percentage of mice showing normal toe spreading reflex (score 0: no toe extension; score 2: full normal extension) after crush. The difference between WT and mutant mice was significant from day 12 to day 70. p < 0.001 (two-way ANOVA), n = 5. (E) Quantification of sensory-motor function in WT and mutants. Note permanent failure of recovery in mutants, although mutant and WT SFIs are similar before and immediately after injury. The difference between WT and mutant mice was significant from day 12 to day 72. Error bars: ± SEM; p < 0.01, n = 4. (F) Increased regeneration in *Wld*^s^ nerves infected with c-Jun adenovirus compared to GFP control virus, shown by galanin immunolabeling of longitudinal sciatic nerve sections 3 days after crush. (G and H) Regeneration failure in *Wld*^s^ nerves can be rescued to WT levels by enforced c-Jun expression in Schwann cells. (GFP) nerves infected with GFP control adenovirus; (c-Jun) nerves infected with c-Jun adenovirus; the WT and *Wld*^s^ genotypes are indicated. Regeneration is measured by the nerve pinch test in (G) and by counting galanin^+^ axons 3 mm from the crush site (H). Error bars: ± SEM; ^∗^p < 0.05, ns = not significant), n = 4. See also Figures S2 and S6.

In WT and c-Jun mutants, sciatic nerve crush abolished the response to toe pinching and decreased the responses to light touch and heat to the minimum measurable by these assays. Control mice recovered in 3–4 weeks as expected, using the toe-pinching test. c-Jun mutants, however, showed minimal recovery, even after extensive (up to 70 day) periods (Figure 7A). Similarly, even 70 days after crush mutant mice showed no recovery in the Hargreaves and Von Frey tests, although control mice recovered fully (Figures 7B and 7C).

To measure motor function we used the toe-spreading reflex. We found that toe extension, abolished by nerve crush, recovered on schedule in WT controls but failed to recover even at 70 days in mutants (Figure 7D).

To measure sensory motor coordination we measured the sciatic functional index (SFI; Inserra et al., 1998). We found the expected reduction in SFI following sciatic nerve crush in WT and c-Jun mutants, but a permanent failure of recovery in mutants only (Figure 7E). These experiments show that Schwann cell c-Jun is a necessary driver of functional recovery of injured peripheral nerves.

### c-Jun Is Sufficient to Generate a Growth-Supportive Nerve Phenotype

Having shown that c-Jun activation is necessary for the conversion of injured nerves to an environment that supports repair, we tested whether c-Jun activation alone was sufficient for this critical transformation.

We took advantage of *Wld*^*s*^ mice, in which axons degenerate slowly after cut, and Schwann cells therefore remain differentiated and unsupportive of repair (Coleman and Freeman, 2010). We confirmed delayed regeneration in these mice compared to WT controls using the nerve pinch test and counting galanin-positive fibers distal to nerve crush (Figures 7F and 7H). Previously we showed that Schwann cells in *Wld*^*s*^ nerves fail to activate c-Jun after injury (Jessen and Mirsky, 2008). We therefore used adenoviral infection to enforce c-Jun expression in crushed *Wld*^*s*^ nerves (Figure S6). Remarkably, this converted the inhospitable *Wld*^*s*^ nerves to a terrain that supported regeneration as effectively as WT nerves (Figures 7F–7H).

This shows that c-Jun is not only necessary but also sufficient for the generation of a growth supporting environment in injured nerves. This observation also confirms that regeneration failure in *Wld*^*s*^ mice is caused by a failure of timely Schwann cell injury response in these animals.

## Discussion

Identification of transcription factors that define cell type, control transit between differentiation states, and enable tissues to repair is a central issue in regenerative biology. The response of Schwann cells to injury provides an exceptionally striking example of a phenotypic transition by adult, differentiated cells. This process is also the basis for the singular regenerative power of peripheral nerves. We show that the Schwann cell injury response represents a c-Jun dependent natural reprogramming of differentiated Schwann cells to generate the repair cell, a distinct Schwann cell state (Bungner cell, since they form Bungner’s bands) specialized to promote regeneration. In mice without c-Jun in Schwann cells, activation of the repair program fails. The disregulated repair cell formed in these mutants is unable to support normal axonal regeneration, neuronal survival, myelin clearance, and macrophage activity. The result is a striking failure of functional recovery.

In addition to the c-Jun mutant, *Wld*^*s*^ mice represent another situation where Schwann cell c-Jun is not activated during attempted regeneration after injury (Jessen and Mirsky, 2008). In this case also, the result is substantial reduction in regeneration. We find that enforced c-Jun expression in injured *Wld*^*s*^ nerves is sufficient to restore axonal regeneration rates to WT values, lending significant support to our model.

It is important to note that although the Bungner cells generated in the mutants are dysfunctional, other Schwann cell functions are normal. Thus, mutant cells remyelinated those axons that regenerated, Schwann cell development appeared normal, and Schwann cells and nerve function in uninjured adults were normal. Although 172 genes were disregulated in the distal stump of the c-Jun mutants, the large majority of the ∼4,000 genes regulated in injured WT nerves remained normally regulated. Therefore, the absence of c-Jun does not have a general impact on the Schwann cell phenotype. Instead, c-Jun appears to have a specific function in adult cells, where it is required for activation of the repair program and timely suppression of the myelin program.

### The Schwann Cell Injury Response Resembles Transdifferentiation

The Schwann cell response to injury is commonly referred to as dedifferentiation, implying that adult denervated cells revert to an earlier stage resembling the immature Schwann cells of perinatal nerves (Harrisingh et al., 2004; Jessen and Mirsky, 2005; Chen et al., 2007; Woodhoo et al., 2009). It is becoming clear, however, that this view is incomplete. These cells have a different structure, molecular profile, and function. Therefore, the immature cells, generated from Schwann cell precursors during development, and Bungner cells generated in response to adult nerve injury, represent two distinct differentiation states.

In injured nerves, myelinating Schwann cells, that are specialized to support fast conduction of action potentials, transform to Bungner cells that are specialized for the unrelated task of organizing nerve repair. This represents an unambiguous change of function, brought about by the combination of dedifferentiation and activation of an alternative differentiation program, the c-Jun dependent Schwann cell repair program. Transitions that share this set of features have been described in other systems, where they are generally referred to as transdifferentiation (Jopling et al., 2011).

### c-Jun Is a Global Regulator of a Schwann Cell Repair Program

The regeneration defects in the c-Jun mutant are substantially more severe than those reported for other mouse mutants, in spite of the fact that the genetic defect is restricted to Schwann cells. The likely reason is the number and diversity of the molecules controlled by this single transcription factor. Among the 172 molecules that are abnormally expressed in the mutant are growth factors, adhesion molecules, growth-associated proteins, and transcription factors. This allows c-Jun to integrate a broad collection of functions that support nerve regeneration, and therefore to act as a global regulator of the Schwann cell repair program. This program involves regulation of molecules that have been directly implicated in repair such as the surface proteins N-cadherin, p75NTR, and NCAM, and the signaling molecules GDNF, artemin, sonic hedgehog, and BDNF. It also includes the morphogenetic processes that change myelinating cells to process bearing cells forming regeneration tracks, and the conversion of Schwann cells to cells equipped to rapidly clear myelin from injured nerves (Stoll et al., 2002; Chen et al., 2007; Vargas and Barres, 2007; Wang et al., 2008; Gordon et al., 2009; Höke and Brushart, 2010; Angeloni et al., 2011).

The exceptional repair potential of peripheral nerves is likely due to the coordinated functions of the repair program. Yet individual factors can also be presumed to play a prominent role, as exemplified by the enhanced regeneration seen when GDNF and artemin levels are increased in c-Jun mutant facial nerves (Fontana et al., 2012).

### Activation of c-Jun and Interaction with Other Transcriptional Regulators

c-Jun is absent from Schwann cell precursors, expressed in immature cells in vivo and in cultured Schwann cells, suppressed by Krox-20 on myelination, but rapidly re-expressed at high levels in Schwann cells of injured nerves (Parkinson et al., 2004, 2008; D.K.W., unpublished).

Among potential intracellular activators of c-Jun is the AP-1 transcription complex, of which c-Jun is a key component. AP-1 activity, in turn, is controlled by numerous signals, including the major MAPK pathways Erk1/2, JNK, and p38. These are all activated in injured nerves and therefore potential upstream regulators of c-Jun (Sheu et al., 2000; Myers et al., 2003; Harrisingh et al., 2004; Jessen and Mirsky, 2008: Parkinson et al., 2008; Napoli et al., 2012; Yang et al., 2012). Genetically, the transcription factor Sox2 is not downstream of c-Jun, since Sox2 remains normally upregulated in injured c-Jun mutant nerves (Figure S4).

We described previously that c-Jun shows cross-inhibitory interactions with the pro-myelin transcription factor Krox20 (Parkinson et al., 2008). Mirroring the function of c-Jun in denervated cells, Krox20 is involved in the regulation of 100–200 genes in myelinating Schwann cells (P. Topilko, personal communication) and is required for the normal activation of the myelin program. We therefore suggest that Krox20/c-Jun are central components of a cross-inhibitory switch that regulates cell fate in injured and regenerating nerves.

### Macrophage Recruitment and Activation

The long term persistence of Schwann cell lipid droplets and large multivacuolated (foamy) macrophages in transected mutant nerves suggests problems with lipid clearance and macrophage activation and exit. Recent evidence indicates that failure of lipid breakdown may delay regeneration (Winzeler et al., 2011).

The reduced macrophage numbers in the mutant early after injury is unlikely to contribute substantially to the regeneration problems, a conclusion supported by the microfluidic chamber experiments, where axon growth fails in the presence of mutant Schwann cells, even in the absence of macrophages. Even severe depletion of invading macrophages has no effect on the number of myelinated axons in dorsal roots following nerve injury (Barrette et al., 2008).

### There Is Extensive Death of Injured Neurons in c-Jun Mutants

The disregulated mutant Bungner cell not only fails to support axon regeneration, but also fails to rescue injured neurons from death. In the mutants, injured type B DRG neurons are about twice as likely to die as in WT mice. Even more notable is the death of about a third of type A neurons, because we find no death of these cells in WT animals, in agreement with previous work in mice and other species (Jiang and Jakobsen, 2004). The majority of facial motoneurons also die after facial nerve injury in the mutant (Fontana et al., 2012).

### The Identity of the Bungner Repair Cell

The observation that denervated adult Schwann cells acquire the ability to generate melanocytes, a property of Schwann cell precursors but not of immature Schwann cells (Adameyko et al., 2009), raises an intriguing possibility. Namely that after injury, Schwann cells dedifferentiate past the immature Schwann cell stage to a cell that shares some properties in common with the Schwann cell precursor.

c-Jun is not significantly expressed in Schwann cell precursors (D.K.W., unpublished). It is therefore possible that the unique identity of the Bungner repair cell in adult nerves consists of a c-Jun-activated repair program in a cell that in significant other aspects has dedifferentiated more completely than hitherto envisaged.

It is clear that the transdifferentiation of myelinating cells to Bungner cells is central to nerve repair. But much remains to be learned about the twin components of this process, the dedifferentiation and repair programs, and about the molecular links that integrate them. This includes issues of practical importance such as the identification of methods to sustain expression of the repair program over the long periods required for nerve repair in humans, and the question of whether the repair program can be activated in other glial cells.

## Experimental Procedures

Animal experiments conformed to UK Home Office guidelines. *P*_*0-*_*CRE*^*+*^*/c-Jun*^*fl/fl*^ mice were generated as described (Parkinson et al., 2008). *P*_*0-*_*CRE*^−^*/c-Jun*^*fl/fl*^ littermates were used as controls. *c-Jun* was excised from *c-Jun*^*fl/fl*^ cells using adenovirally expressed *CRE-*recombinase. Experiments for which n numbers are not shown in figure legends were done at least three times.

### Nerve Injury

Sciatic nerves of adult mice were cut or crushed at the sciatic notch.

### Microarray Hybridization

RNA was extracted, cDNA generated and applied to Mouse 430 2.0 array (Affymetrix, MA). Significantly different genes were selected with Bayes’ t test. After control for false discovery rate, genes with a p value of less than 0.05 were filtered out. The microarray data are MIAME compliant.

### In Situ Hybridization

This was performed as described (Lee et al., 1997).

### RT-QPCR

QPCR was performed with Sybrgreen SYBR Green JumpStart (Sigma) and carried out using Chromo4 Real Time Detector (Bio-Rad). For primers see Table S5. Data was analyzed using Opticon monitor 3 software and fold-changes determined with the Livak method (see Supplemental Information).

### Adenoviral Infection

Adenovirus expressing *c-Jun* was generated by cloning mouse *c-jun* sequence into pAdTrack-CMV. This was recombined into adenoviral backbone plasmid pAdEasy-1 in bacteria. Schwann cell cultures (Dong et al., 1999) were infected with purified adenoviral supernatants (Parkinson et al., 2001, 2008).

### Immunocytochemistry

Nerve segments, spinal cords or Schwann cell cultures were fixed in paraformaldehyde (PF)/PBS for 10 min–2 hr. Sections were fixed in 2% or 4% PF/PBS for 10 min or methanol for 30 min prior to immunolabeling. Alternatively, nerves were fixed in PF/PBS for 24 hr and wax embedded. Four micrometer sections were deparaffinized and antigen retrieved prior to immunolabeling. Blocking solution was used before incubation with primary antibodies overnight followed by secondary antibodies for 30 min to 1 hr. The first layer was omitted as a control.

### Functional Tests

The nerve pinch test was used to assess axonal regeneration distance in vivo. Sensory motor coordination was assessed using mouse footprints to calculate the sciatic functional index. Sensory function was assessed by Von Frey Hair analysis, the Hargreaves test and response to toe pinching. Motor function was analyzed by observing toe spread (see Supplemental Information).

### Retrograde Tracer Labeling

True Blue (2 μl) was injected into the tibialis anterior muscle at three sites to label motor neurons in spinal cord segments L2 to L6. Seven days later, mice were perfused. Serial 30 μm sections were collected and the number of labeled neurons was counted (Supplemental Information).

### Counts of DRG Neurons, Schwann Cells, and Macrophages

The L4 DRG was cryosectioned. DRG neurons (nuclei) were counted as described (Puigdellívol-Sánchez et al., 2000). Ten micrometer serial sections were labeled with Neurotrace fluorescent Nissl green stain. Every sixth section was analyzed and systematic random sampling (SRS; see Supplemental Information) applied to ensure unbiased estimation of neuron numbers. A and B cells were differentiated on size and morphological criteria as described (Tandrup et al., 2000). For further confirmation, A cells in 10 week cut WT and mutant DRG were quantified by nucleolar counts (Jiang and Jakobsen, 2010). Both nuclear and nucleolar counts were corrected as described in Abercrombie (1946). Schwann cells and macrophages in injured tibial nerves were counted in whole transverse sections in the electron microscope using SRS (see Supplemental Information).

### Lipid Labeling

Following PF fixation, 10 μm sections were treated with 2% OsO_4_-PBS solution overnight. Percentage stained nerve area relative to that in uninjured nerves was quantified using NIH ImageJ.

### Western Blotting

Frozen nerve samples or cell lysates were blotted as described (Parkinson et al., 2004).

### Microfluidic Chambers

Using a three-compartment microfuidic chamber (Taylor et al., 2005), 5,000 adult DRG neurons were plated in the central compartment in defined medium with 50 mM glucose (Dong et al., 1999). 2 × 10^5^ WT Schwann cells, *c-Jun* null cells or *c-Jun* null cells infected with c-Jun adenovirus were plated in the side chambers. The number of axons longer than 50 μm growing into the side compartment was counted. Alternatively the area of axonal elongation into the side chambers from the microgrooves up to 300 μm beyond them was measured using ImageJ.

### Ultrathin Sections

#### Thickness of Myelin Sheath (g Ratio)

Photographs (15–20) were taken at 2500× of transverse ultrathin sections of tibial nerve 5 mm from the sciatic notch. The g ratio was calculated for each myelinated fiber (axon diameter divided by diameter of the axon and myelin sheath). Statistical difference was measured using Mann Whitney U test.

#### Counts of Myelinated Axons in Adult Tibial Nerve and Dorsal and Ventral Roots after Nerve Crush or Cut

A montage of ultrathin sections (×1000) was made and the number of myelinated fibers counted. For unmyelinated fibers, 30%–40% of each nerve was photographed (×5000). The ratio myelinated:unmyelinated fibers was measured and the total for each nerve/root was multiplied by total myelin fiber count, as described elsewhere (Coggeshall et al., 1997). For counts of Schwann cells and macrophages, ultrathin sections were mounted on film, nuclei counted in every third field and multiplied by 3 to generate totals. Statistical difference was measured using Mann Whitney U test.

### Migration Assays

Macrophage migration was assessed using 6.5 mm Transwells with 5 μm pores (Corning Costar; see Supplemental Information).

### Statistical Analysis

Data are presented as arithmetic mean ± standard error of the mean (SEM) unless otherwise stated. Statistical significance was estimated by Student’s t test, two-way ANOVA, or Mann-Whitney U-test.

## Supplemental Information

Document S1. Figures S1–S6, Tables S1, S4, and S5, and Supplemental Experimental Procedures

Table S2. 172 Genes that Are Differentially Regulated between WT and c-Jun Mutant Sciatic Nerve at 7 Days after Nerve Transection The 172 genes that are disregulated in the distal stump of c-Jun mutant nerves (cKO) compared to the distal stump of WT nerves (cKO/WT). The data were obtained by microarray 7 days after transection without regeneration. Red type indicates genes expressed at higher levels in cut c-Jun mutant nerves compared to cut WT nerves (66 genes), while blue type indicates those genes that are expressed at lower levels in cut c-Jun mutant nerves compared to cut WT nerves (106 genes).

Table S3. Expression Levels (QT-RT-QPCR) of 33 of the 172 Differentially Regulated Genes (Determined by Microarray Analysis) in Uninjured and 7 Day Cut Nerves QT-RT-QPCR analysis of the regulation of the gene subset shown in Figure 1D. The table shows expression levels in cut relative to uncut nerves in mutant and WT mice and shows statistical significance of each comparison. Error bars ±SEM; (n = 3–6 pools of animals with 3 animals in each pool; see figure legend).
