# Supplementary material for: c-Jun Reprograms Schwann Cells of Injured Nerves to Generate a Repair Cell Essential for Regeneration
Source: Neuron. 2012 Aug 23;75(4):633–47. doi: 10.1016/j.neuron.2012.06.021 (PMC3657176; doi:10.1016/j.neuron.2012.06.021)
Supplement: Document S1. Figures S1–S6, Tables S1, S4, and S5, and Supplemental Experimental Procedures [file mmc1.pdf]

**Neuron, Volume *75***

# Supplemental Information

# c-Jun Reprograms Schwann Cells of Injured Nerves to Generate a Repair Cell Essential for Regeneration

Peter J. Arthur-Farraj, Morwena Latouche, Daniel K. Wilton, Susanne Quintes, Elodie Chabrol, Ambily Banerjee, Ashwin Woodhoo, Billy Jenkins, Mary Rahman, Mark Turmaine, Grzegorz K.Wicher, Richard Mitter, Linda Greensmith, Axel Behrens, Gennadij Raivich, Rhona Mirsky, and Kristján R. Jessen

**Inventory of Supplemental Information**

1. **Supplemental Fig 1:** This figure is linked to main figure 1. Here we show that the thickness of the myelin sheath as denoted by the g-ratio is similar in the peripheral nerves of WT and c-Jun mutant mice.

2. **Supplemental Fig 2:** This is linked to main figures 1, 4, 5 and 7. This figure shows that c-Jun is specifically ablated from Schwann cells and not from other components of the peripheral nervous system such as sensory neurons, motor neurons, macrophages or fibroblasts.

3. **Supplemental Fig 3:** This is linked to main figures 1 and 6. Here we show that axon disintegration following nerve injury is not regulated by Schwann cell c-Jun and occurs on schedule in mutant mice.

4. **Supplemental Fig 4:** This figure is linked to main figure 1 and shows that Sox2, another negative regulator of myelination, is expressed normally in the nerves of c-Jun mutant mice following nerve transection.

5. **Supplemental Fig 5:** This is linked to main figure 6. (A and B) provide additional information about the slower loss of myelin proteins in the nerves of c-Jun mutant mice following injury. (C) shows that macrophages are recruited to the nerve more slowly after injury in c-Jun mutant mice. (D) shows that conditioned medium generated by culturing nerves from c-Jun mutant mice is less able to stimulate macrophage migration than that generated from the nerves of WT mice. (E) shows that levels of the mRNA transcripts for several major cytokines are similar in WT and c-Jun mutant nerves 36hr post transection.

6. **Supplemental Fig 6**: This is linked to main figure 7. Here we show GFP expression in Schwann cells following injection of the c-Jun adenovirus into the tibial nerve.

7. **Supplemental Table 1:** This is related to main figure 1 and shows that there are a similar number of Remak bundles and myelinating Schwann cells in adult nerves from WT and c-Jun mutant mice.

8. **Supplemental Table 2:** This is related to main figure 1 and provides additional information about the 172 genes that are differentially regulated between WT and c-Jun mutant nerves 7 days after transection.

9. **Supplemental Table 3:** This is related to main figure 1. It shows that the genes in figure 1,D are abnormally regulated in cut nerves of c-Jun mutant nerves compared to their regulation in WT nerves, and that these differences are statistically significant.

10. **Supplemental Table 4:** This is related to main figure 2 and shows quantification of the comparative QPCR of *olig1*, *GDNF*, *Shh* and *GAPDH* mRNA from uncut adult sciatic nerve, adult sciatic nerve 7 days after cut and E18 sciatic nerve.

11. **Supplemental Table 5:** This is linked to main figures 1 and 2 and shows the primers used for QPCR analysis.

12. **Supplemental Experimental Procedures**

**Supplemental Fig. 1. g-ratios of control and c-Jun mutant adult nerves**

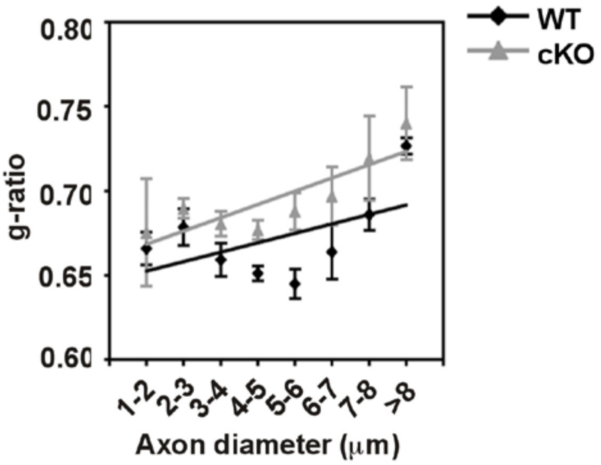


<u>Supplemental Figure Legend 1</u>

Myelin sheath thickness measured by g-ratio (diameter of axon/diameter of axon plus sheath) for different sizes of axon in the tibial nerve of c-Jun mutant (cKO) and control (WT) mice, n=3. The mean g-ratio for each axonal size category is shown together with linear regression lines. Error bars show the standard deviation of the mean. Although the g-ratios for the control mice appear slightly higher than for the c-Jun mutant mice, the differences in g-ratio for every axonal category were not significant using the Mann-Whitney U test.

**Supplemental Fig. 2. In c-Jun mutant mice, c-Jun expression is normal in motoneurons, DRG neurons, macrophages and fibroblasts**

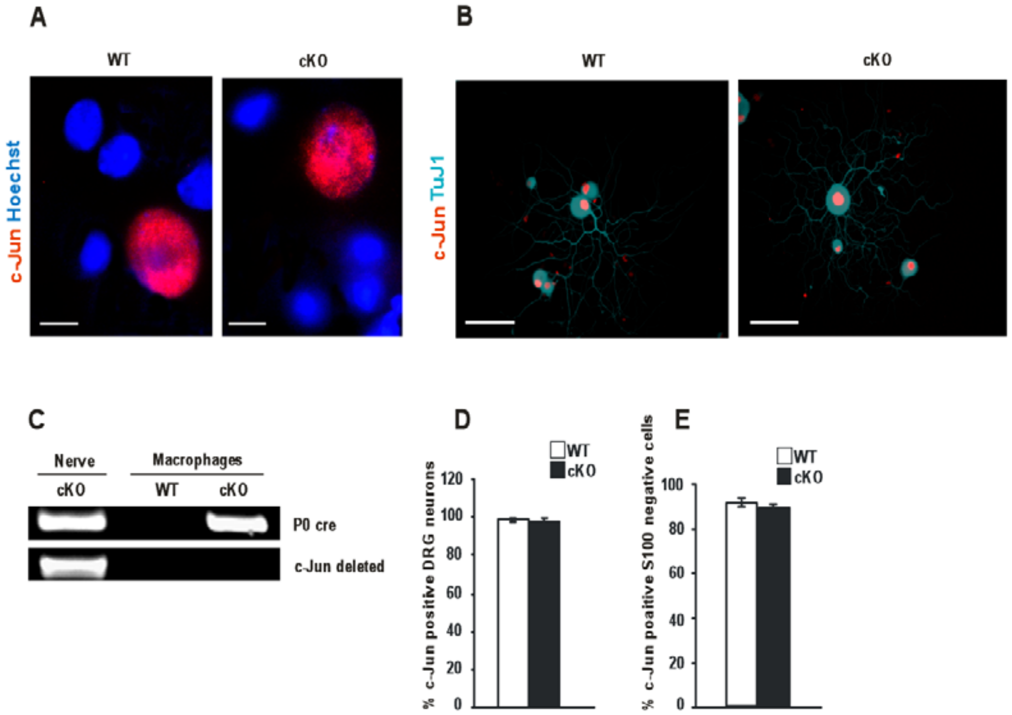


Supplemental Figure Legend 2

(A) Fluorescence micrograph showing c-Jun immunolabeled motoneurons in the L2 to L6 region of the spinal cord of both control (WT) and mutant (cKO) mice 7 days after nerve crush. Bar: 25mm. (B) Fluorescence micrograph showing c- Jun immunolabeling in purified DRG neuron cultures of both mutant (cKO) and control mice (falsely coloured to enable c-Jun staining to be clearly visualised). Bar: 100mm (C) PCR of DNA from macrophage and nerve extracts showing that while both possess the *P0cre* transgene the deleted *jun* band (Jun del) which arises upon excision of the *c-jun* gene can only be detected in the nerve extract. (D) Similar numbers of DRG sensory neurons express c-Jun in cultures derived from control (WT) and mutant (cKO) mice. (E) Mouse sciatic nerve cultures from controls (WT) and mutants (cKO) contain similar numbers of c-Jun expressing fibroblasts, n=3 (i.e S100 negative flattened cells with large nuclei, judged by Hoechst staining). Error bars represent SEM.

**Supplemental Fig. 3. The rate of axonal degeneration after nerve transection is normal in c-Jun mutant mice.**

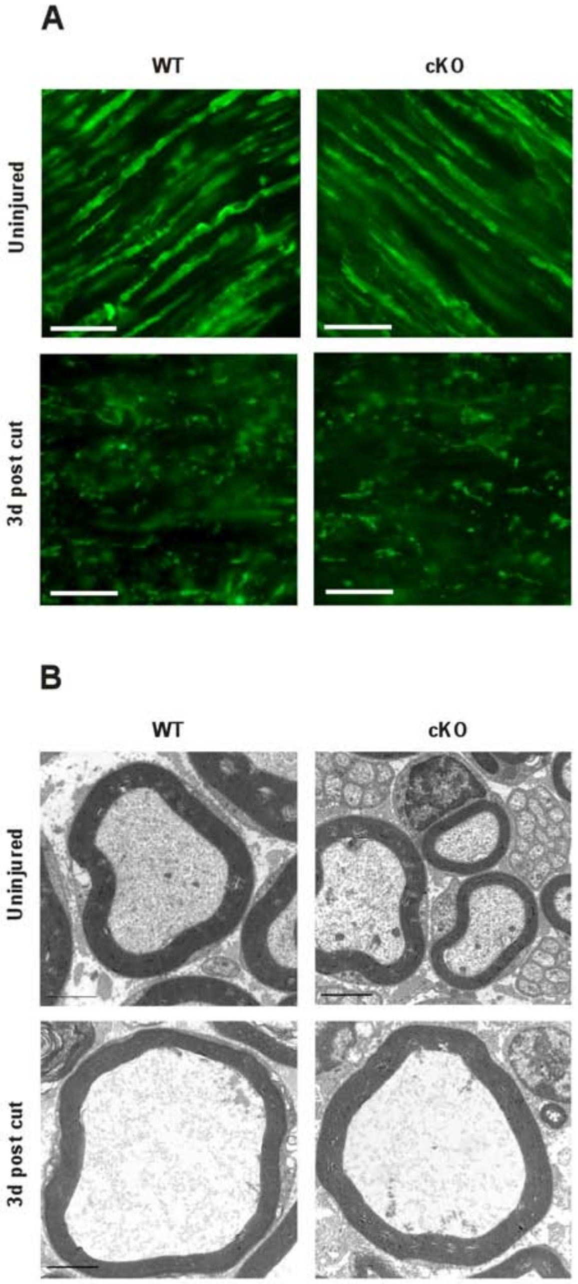


Supplemental Figure Legend 3

(A) Immunolabeling for β-tubulin (TUJ1) in c-Jun mutant (cKO) and control (WT) nerves before and 3 days after sciatic nerve cut. Note the similar broken pattern of labeling 3 days after cut in both nerves. Bar: 100μm.

(B) Electron micrographs showing axons in c-Jun mutants (cKO) and control (WT) animals before (uninjured) and 3 days after sciatic nerve cut. Note similar loss of axonal contents, including mitochondria and microtubules, in both nerves after injury. Bar: 2μm.

**Supplemental Fig. 4. In the c-Jun mutant, Sox2 is normally upregulated in Schwann cells of injured nerves**

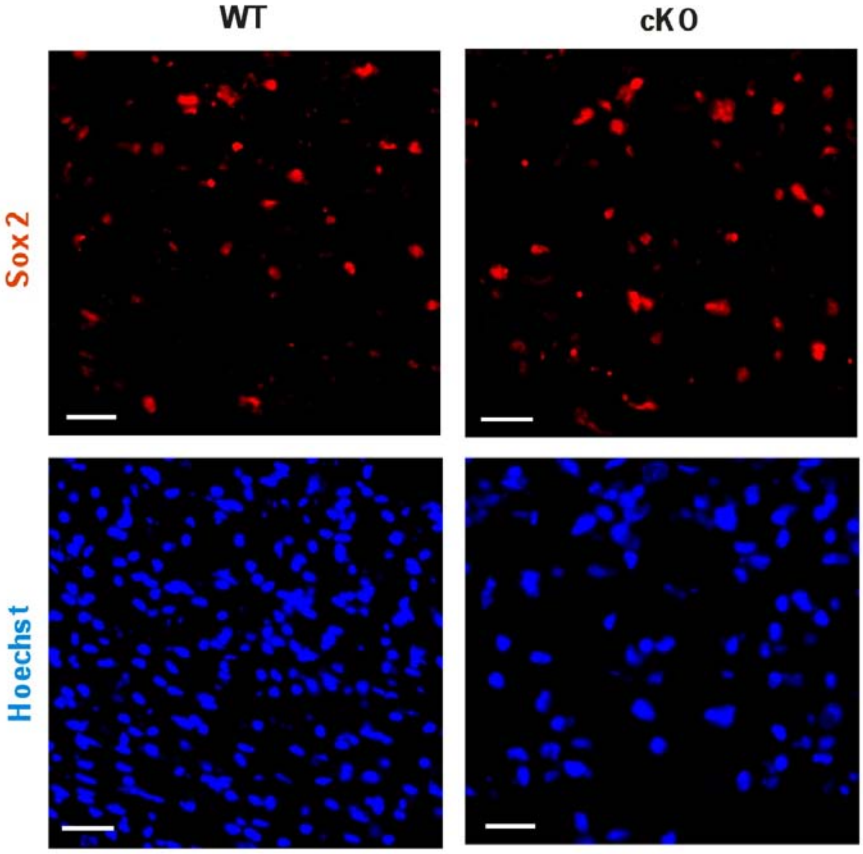


Supplemental Figure Legend 4

Fluorescence micrograph showing Sox 2 immunolabeled cells in 7 day cut mutant (cKO) and control (WT) nerves. Bar: 50µm.

**Supplemental Fig. 5. MBP immunoreactivity in injured nerves, macrophage migration and cytokine expression: a comparison between c-Jun mutant and WT mice.**

cKO nerves show delayed loss of MBP immunoreactivity after injury

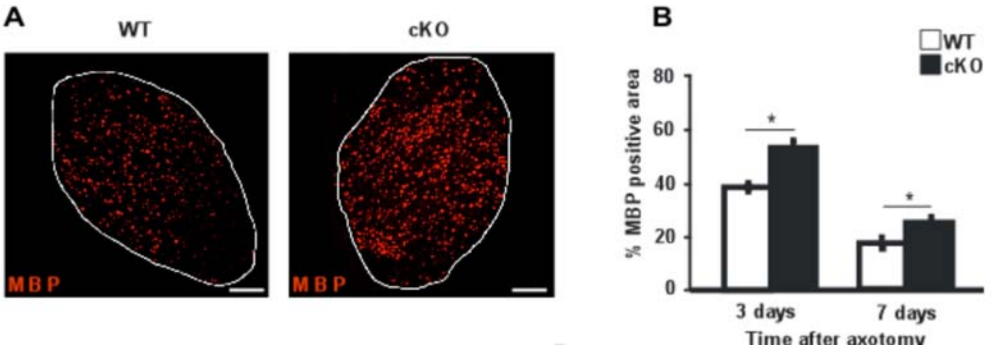


Macrophage numbers in WT and cKO nerves

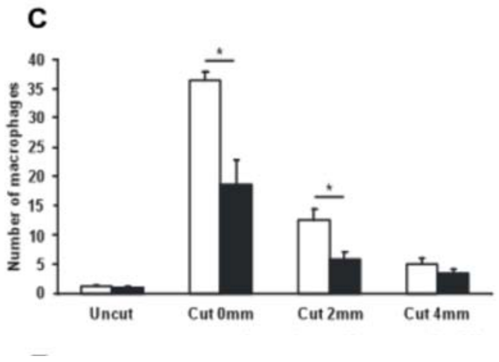


Macrophage migration assay

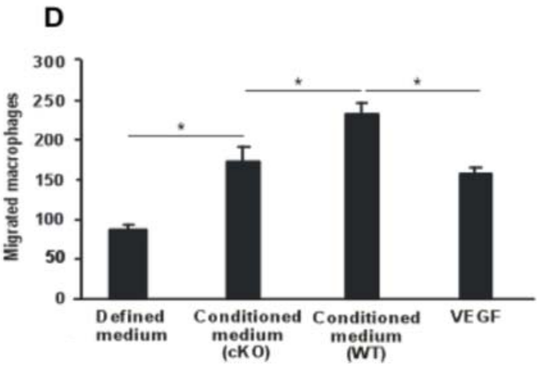


Cytokine expression in cKO nerves relative to WT at 36hr

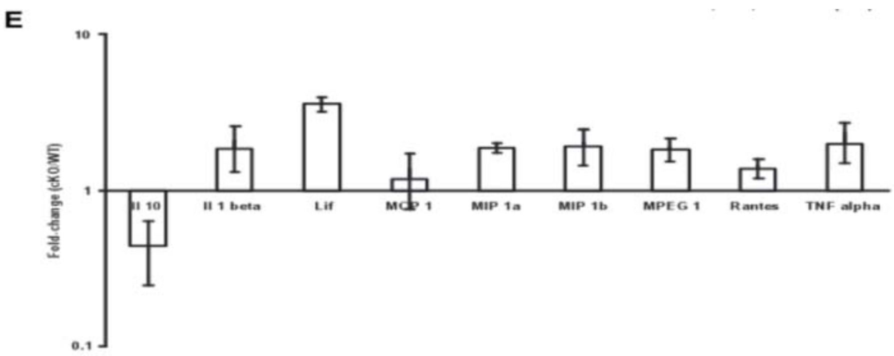

Supplemental Figure Legend 5

(A) MBP immunolabeling of adult mutant (cKO) and WT sciatic nerves 4 days after transection. Bar: 50 µm. (B) Quantification of (A) ($p<0.05$), n=3. (C) Counts of macrophages (immunolabeled with integrin αM chain) in uncut and transected mutant (cKO, black columns) and WT (white columns) sciatic nerves 3 days after injury. Fewer macrophages are present in the mutant both at the cut site and 2mm distal from it ($p< 0.05$), n=3. (D). Peritoneal macrophage migration in the presence of defined medium, conditioned medium from WT and (cKO) sciatic nerves and the chemoattractant VEGF (10ng/ml). Conditioned medium from mutant sciatic nerves attracts fewer macrophages than medium from WT nerves ($p<0.05$), n=3. (E) Expression of major cytokines in the distal stump of mutant nerves 36h post-transection determined by RT-QPCR. The data are expressed as fold change in the mutant compared to WT compared to control, n=2. Error bars represent SEM.

**Supplemental Fig. 6. In vivo infection of sciatic nerves with c-Jun adenovirus results in widespread expression of the virus in Schwann cells.**

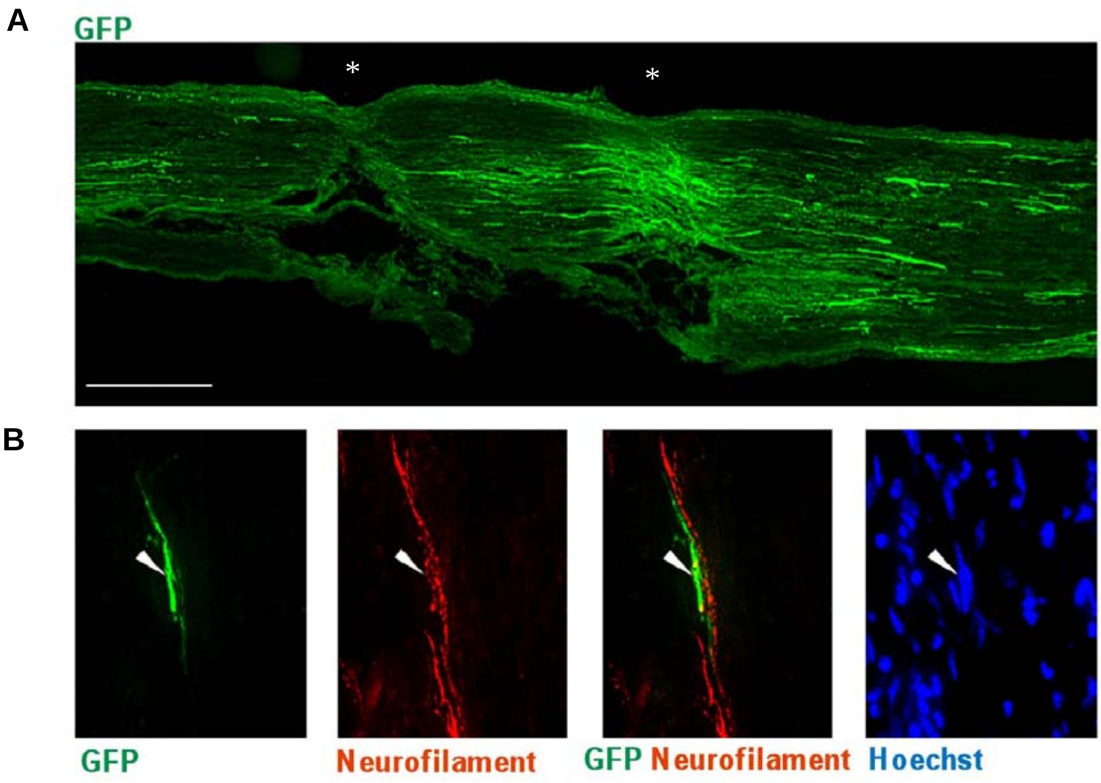


Supplemental Figure Legend 6

(A) GFP immunolabeling revealing the extent of c-Jun adenovirus expression in $Wld^s$ infected nerves 6 days after viral injection. (* indicates disruption to tissue and immunolabelling due to nerve pinch). (B) A Schwann cell infected with the c-Jun adenovirus identified by GFP autofluorescence. The cell is closely associated with a regenerating axon (galanin +ve, in red) in a regenerating $Wld^s$ nerve 6 days after infection and 3 days after crush. Bar: 0.5mm

**Supplemental Table 1**

Number of myelinating Schwann cells and Remak bundles in WT and c-Jun mutant adult nerves

| Tibial nerve | Remak bundles | Schwann cell nuclei |
|---|---|---|
| Control (n=4) | 614±62 | 93.5±6.91 |
| c-Jun mutant (n=4) | 538±24.5 | 97.3±4.44 |

<u>Supplemental Table Legend 1</u>

Counts are ± SEM. Differences in Remak profiles are not statistically different between control and c-Jun mutant nerves. For Remak cells and Schwann cell nuclei $p>0.05$ (Mann-Whitney U test).

**Supplemental Table 2**

172 genes that are differentially regulated between WT and c-Jun mutant sciatic nerve at 7 days after nerve transection

See excel file.

<u>Supplemental Table Legend 2</u>

The 172 genes that are disregulated in the distal stump of c-Jun mutant nerves (cKO) compared to the distal stump of WT nerves (cKO/WT). The data were obtained by microarray 7 days after transection without regeneration. Red type indicates genes expressed at higher levels in cut c-Jun mutant nerves compared to cut WT nerves (66 genes), while blue type indicates those genes that are expressed at lower levels in cut c-Jun mutant nerves compared to cut WT nerves (106 genes).

**Supplemental Table 3**

Expression levels (QT-RT-QPCR) of 33 of the 172 differentially regulated genes (determined by microarray analysis) in uninjured and 7 day cut nerves

See excel file.

<u>Supplemental Table Legend 3</u>

QT-RT-QPCR analysis of the regulation of the gene subset shown in Fig.1D. The table shows expression levels in cut relative to uncut nerves in mutant and WT mice and shows statistical significance of each comparison. Error bars +/-SEM; (n=3-6 pools of animals with 3 animals in each pool; see figure legend).

**Supplemental Table 4**

Comparative QPCR of *olig1*, *GDNF*, *Shh* and *GAPDH* mRNA from uncut adult sciatic nerve, adult sciatic nerve 7 days after cut and E18 sciatic nerve, all from WT mice.

| Gene | Adult uncut (CT) | Adult 7 day cut (CT) | E18 (CT) |
|---|---|---|---|
| *Olig1* | ND | 30.72 ± 0.88 | ND |
| *GDNF* | ND | 28.51 ± 0.03 | 35.48 ± 1.77 |
| *SHH* | 33.09 ± 2.2 | 27.15 ± 2.57 | ND |
| *GAPDH* | 20.09 ± 2.14 | 19.64 ± 1.31 | 18.27 ± 2.10 |

<u>Supplemental Table Legend 4</u>

The table shows the cycle threshold (CT) values for *olig1, GDNF*, *SHH* and *GAPDH* in uncut WT adult mouse sciatic nerve, adult sciatic nerve distal stump 7 days after transection and in embryonic day (E) 18 nerve. Note that all these genes were strongly upregulated following injury, while they were either not detectable (ND) (*olig1, SHH)* or barely detectable (*GDNF)* in immature Schwann cells (E18). These cDNAs were run on the agarose gel shown in Figure 2 E.

## Supplemental Table 5

Primers used for QPCR

| Gene symbol | Forward primer | Reverse primer | Reference |
|---|---|---|---|
| Cdh1 | CAGGTCTCCTCATGGCTTTGC | CTTCCGAAAAGAAGGCTGTCC | 1 |
| Fabp7 | GGACACAATGCACATTCAAGAAC | CCGAACCACAGACTTACAGTTT | 1 |
| Lrrtm4 | CTGCTGCTGGTTATGCTGAC | CCAGAAATGTTCTCAGGGATGTC | 1 |
| Adam23 | CGTCCTTCTCCTGCTACCTG | TGATAGGGGCTTTCTGAGTCC | 1 |
| Lrrn3 | GATTGCCCCCAATTATGTACCTG | GGAGCAGAATCTGTGTGTCGG | 1 |
| Gpr37l1 | GGGCTGCCACCTCATCTTTAG | GATGGATGGGCTTGGGATATTC | 1 |
| Kcnj10 | GTCGGTCGCTAAGGTCTATTACA | GGCCGTCTTTCGTGAGGAC | 1 |
| Cmtm5 | TGGTCTCCGTCTTTGCCTATG | CTCAGTGGTACTGGGCATCAG | 1 |
| Tyrp1 | CCCCTAGCCTATATCTCCCTTTT | TACCATCGTGGGGATAATGGC | 1 |
| Sostdc1 | AGCTCCTTCAGAGGGCTGAT | GAGGCAGGCATTTCAGTAGC | 2 |
| Mbp | AATCGGCTCACAAGGGATTCA | TCCTCCCAGCTTAAAGATTTTGG | 1 |
| Chl1 | ATGAAAAACCTCTCAAGGGAAGC | TGCGCCAATAAACGATCCATC | 1 |
| Mpz | CGGACAGGGAAATCTATGGTGC | TGGTAGCGCCAGGTAAAAGAG | 1 |
| Plat | TGACCAGGGAATACATGGGAG | CTGAGTGGCATTGTACCAGGC | 1 |
| Bcl2 | GTCGCTACCGTCGTGACTTC | CAGACATGCACCTACCCAGC | 1 |
| Fzd8 | ATGGAGTGGGGTTACCTGTTG | CACCGTGATCTCTTGGCAC | 1 |
| Nrcam | AACCTGAGTATGCAGTCGTCC | GTTGGAGTAGGAGCGACAACC | 1 |
| Artn | CCCTAGCTGTTCTAGCCCTG | AGGGTTCTTTCGCTGCACAA | 1 |
| BDNF | TCATACTTCGGTTGCATGAAGG | AGACCTCTCGAACCTGCCC | 1 |
| Jun | CCTTCTACGACGATGCCCTC | GGTTCAAGGTCATGCTCTGTTT | 1 |
| Igfbp3 | CCAGGAAACATCAGTGAGTCC | GGATGGAACTTGGAATCGGTCA | 1 |
| Tnc | ACGGCTACCACAGAAGCTG | ATGGCTGTTGTTGCTATGGCA | 1 |
| Shh | AAAGCTGACCCCTTTAGCCTA | TTCGGAGTTTCTTGTGATCTTCC | 1 |
| Gap43 | TGGTGTCAAGCCGGAAGATAA | GCTGGTGCATCACCCTTCT | 1 |
| Cdk5r1 | CTGTCCCTATCCCCCAGCTAT | GGCAGCACCGAGATGATGG | 1 |
| GDNF | CCAGTGACTCCAATATGCCTG | CTCTGCGACCTTTCCCTCTG | 1 |
| Slc6a1 | GAAAGCTGTCTGATTCTGAGGTG | AGCAAACGATGATGGAGTCCC | 1 |
| Frzb | CACAGCACCCAGGCTAACG | TGCGTACATTGCACAGAGGAA | 1 |
| Chst8 | ATGACCCCACAACTCGGAAC | GGCCTGATGCTGAACTTTATACC | 1 |
| Gzmb | CCACTCTCGACCCTACATGG | GGCCCCCAAAGTGACATTTATT | 1 |
| Clca2 | GGATGCTGGCTCACTCTGAT | CAGACCTGGAGGCTGAGTTT | 2 |
| Rgs8 | GCAGGAACAAAGGCATGAGGA | TGCTTCTTCCGTGGAGAGTCT | 1 |
| Igfbp2 | GCC CCC TGG AAC ATC TCT ACT | TCC GTT CAG AGA CAT CTT GCA | 3 |
| Olig1 | ACCAACGTTTGAGCTTGCTT | GGTTAAGGACCAGCCTGTGA | 4 |


1. Spandidos, A., Wang, X., Wang, H. and Seed, B. (2010). PrimerBank: a resource of human and mouse PCR primer pairs for gene expression detection and quantification. Nucl. Acids Res. 38, D792-9.
2. Cui, W., Taub, D.D. and Gardner, K. (2007). qPrimerDepot: a primer database for quantitative real time PCR. Nucl. Acids Res. 35, D805–D809.
3. Lofqvist, C., Willett, K.L., Aspegren, O., Smith, A.C., Aderman, C.M., Connor, K.M., Chen, J., Hellstrom, A., and Smith, LE. (2009). Quantification and localization of the IGF/insulin system expression in retinal blood vessels and neurons during oxygen-induced retinopathy in mice. Invest. Ophthalmol. Vis. Sci. 50, 1831-7.
4. Lin, T., Xiang, Z., Cui, L., Stallcup, W. and Reeves, S.A. (2006) New mouse oligodendrocyte precursor (mOP) cells for studies on oligodendrocyte maturation and function. J. Neurosci. Methods 157, 187-94.

**Supplemental Table 6**

Number of myelinated axons in ventral roots of WT and mutant mice

| | WT | c-Jun mutant |
|---|---|---|
| Uninjured | 981+/- 79; n=3 | 973+/-106; n=3 |
| 70 days post crush | 956+/-44; n=6, | 942+/-31; n=4, |

Supplemental Table Legend 6

In WT mice, the number of myelinated axons in ventral roots remained unchanged after injury as expected. Unexpectedly this was also the case in the mutants. Therefore Schwann cell c-Jun is not required for the support of injured spinal cord motoneurons, unlike that seen in DRG neurons and in motoneurons in the facial nucleus (Fontana et al. 2012).

**Supplemental Table 7**

Counts of Schwann cells and macrophages (using the electron microscope) in the distal stump of injured (5mm from the injury site) and uninjured nerves.

| 4 day crush tibial | Schwann cell nuclei | Macrophage nuclei |
|---|---|---|
| Control (n=5) | 220.5±24.53 | 72.6±21.07 |
| c-Jun mutant (n=3) | 118±9.56 (p<0.05) | 36±7.55 (ns) |

| 14 day crush tibial | Schwann cell nuclei | Macrophage nuclei |
|---|---|---|
| Control (n=5) | 653.4±79.44 | 123±14.28 |
| c-Jun mutant | 499.3±130.8 (ns) | 87±24.57 (ns) |

| 7 day cut tibial | Schwann cell nuclei | Macrophage nuclei |
|---|---|---|
| Control (n=4) | 286.5±16.9 | 49.5±6.30 |
| c-Jun mutant | 190±19.71 (ns) | 46.3±12.29 (ns) |

| 6 week cut tibial | Macrophage nuclei |
|---|---|
| Control (n=4) | 48.5±3.86 |
| c-Jun mutant | 59±14.11 (ns) |

Supplemental Table Legend 7

Counts of Schwann cell and macrophage nuclei in whole transverse sections of tibial nerves from WT control and c-Jun mutant mice.
The cells were counted in whole sections of the tibial nerve in the electron microscope at 8000x magnification 5mm from the cut or crush site. There is no significant difference in the number of Schwann cells in regenerating nerves 14 days after crush, or cut nerves 7 days after injury. There is no significant difference in the number of macrophages present in the mutant and control nerves at any time point. There are significantly fewer Schwann cells in the mutant 4 days after crush ($p<0.05$). n indicates number of animals. There is no significant difference in the number of macrophages present in the mutant and control nerves at any time point of nerves counted. Counts are ± SEM.

## SUPPLEMENTAL EXPERIMENTAL PROCEDURES

### Antibodies and in situ probes

These were as follows: antibodies to c-Jun (BD Transduction Labs); S100β (Dako); myelin protein zero (Morgan et al., 1991); Sox-2, p75NTR (Millipore); CGRP, galanin (Peninsula); GFP (Invitrogen); IBA1(Wako Chemical); N-Cadherin and GAPDH (Abcam); myelin protein zero (Astexx); β-tubulin (Sigma); β-tubulin III, MBP, SMI-31R (Covance); integrin αM chain (Serotec); β1 integrin (Chemicon); N-CAM and L1 antibodies were gifts from G. Rougon and R. Martini, respectively; and secondary antibodies were from Jackson Immunoresearch (FITC and Cy3 conjugates) and Promega (HRP conjugates). Antibodies were used as previously described (Dong et al., 1999; Raivich et al., 2004; Parkinson et al., 2008). *Shh, GDNF and Olig 1* probes were gifts from C. Tickle, M.Saama, and W.D. Richardson respectively.

### Functional tests

*Nerve pinch.* This was carried out as in Seijffers et al. (2007). Regeneration distance *in vivo* was measured, followed by immunocytochemistry on sections of the sciatic nerve. The average number of CGRP and galanin positive fibres crossing the nerve 2 and 3mm from the crush was derived from three sections 60μm apart. In addition distance between the most distal growth cone and the crush site was measured (Siconolfi and Seeds 2001).

*Sciatic functional index (SFI).* Paw prints were measured to calculate the SFI (Inserra et al., 1998). Hindlimb prints were digitized (Epson Perfection 3100) and measured using the FootPrints program (Jungnickel et al., 2010).

*Toe-spreading reflex.* A score of 0 (no extension) to 2 (full extension) was used as in Siconolfi and Seeds (2001).

*Toe-pinch*. The distal part of the lateral three toes was pinched lightly. Hind limb withdrawal and/or vocalization were taken as positive responses. 3 readings on each paw were collected.

*Von Frey Hair analysis.* Return of sensory function and development of mechanical allodynia was assessed 7 days and 10 weeks after nerve crush as in Vogelaar et al., (2004).

*Hargreaves test.* 7 days and 10 weeks after nerve crush an infra-red beam (Ugo Basile 7371 Plantar Test) was positioned under the hind paw and the time until a reflex withdrawal response was noted.

### Counts of DRG neurons and motoneurons

Systematic random sampling (SRS) for counting of DRG neurons was carried out in the following way. Serial 10 μm sections from an entire DRG were placed on 6 slides in such a way that 60 μm separated each section. One of the 6 slides was chosen at random and every neuron (where the nucleus was clearly present) in every section was counted.

30 μm serial sections were cut through the L2-L6 segments of backfilled spinal cords. Fluorescent backfilled neurons showing clear nuclear profiles, were counted in every section using the method of Baron et al., (1985).

### Counts of Schwann cells and macrophages

Systematic, random sampling of Schwann cells and macrophages (identified by standard ultrastructural criteria (e.g. Reichert et al. 1994) in cut and crushed sciatic nerves was carried out in the following way. In the electron microscope, whole

transverse sections, mounted on film (no grid bars), showing the entire profile of a transversely cut nerve, were counted. All Schwann and macrophage nuclei were counted in every field (at 8000x), (adopting a simple rule of nuclear overlap into left and right hand fields to avoid double counting nuclei) in a line completely crossing the entire section. Counting started at the very top of the section and every other line was counted right to the bottom end of the section. This was done on nerves from 3-4 mice as indicated in the table (n numbers).

**Macrophage, lipid droplet and myelin debris counts and migration assays**
The number of macrophages in the distal stump of transected nerves were analysed 3 days after transection by labelling with antibodies to αM as previously described (Hristova et al. 2010). Alternatively total macrophages were identified and counted at 8000X using electron microscopy of transverse sections of distal stumps of tibial nerve 5mm from the crush site at various times after injury (see above). The number of lipid droplets/macrophage and amount of myelin debris/field was counted in the distal stump using transverse sections of the tibial nerve one month after cut. A randomized grid overlay sampling method was used, counting the number of grid nodes overlying a droplet or myelin debris in electron micrographs (x4000 and x8000). This allowed counting of 60-70% of the transectional nerve profile.
To assess macrophage migration, defined medium or medium conditioned for 3 days with 3x 5mm sciatic nerve pieces excised from control or mutant nerves was added to the lower chamber. LPS-activated peritoneal macrophages were added to the upper chamber and after 3hr at 37°C cells on the upper surface were removed and the number of cells on the bottom surface was counted after staining with Hoechst dye.

**Schwann cell cultures**
Schwann cell cultures were made as described in Dong et al. (1999). After purification, (or purification followed by expansion in defined medium (Dong et al., 1999) containing 0.5% horse serum, 10-20 ng/ml neuregulin and $5x10^{-4}$M dbcAMP), the cells were maintained in defined medium containing 0.5% horse serum. For experiments in microfluidic chambers, defined medium containing 0.5% horse serum and 50mM glucose was used.

**RT-QPCR**
QPCR was carried out using the Chromo4 Real Time Detector (Biorad). For primers used see Supplemental Table 5. PCR was performed in 25μl reactions containing 2.5pmoles of each primer, 0.625 units Taq DNA polymerase, 10mM Tris-HCl, 50mM KCl, 3.5mM $MgCl_2$, 0.2mM dNTP stabilizers, 1x reference dye and 62.5ng cDNA. Primer sequences were obtained from Primer Bank and have been extensively tested by real-time PCR experiments for specificity and efficiency.

## SUPPLEMENTAL REFERENCES


Baron, R., Janig, W., McLachlan, E.M. (1985). The afferent and sympathetic components of the lumbar spinal outflow to the colon and pelvic organs in the cat. III. The colonic nerves, incorporating an analysis of all components of the lumbar prevertebral outflow. J. Comp. Neurol. 238: 158-68.

Hristova, M., Cuthill, D., Zbarsky, V., Acosta-Saltos, A., Wallace, A., Blight, K., Buckley, S.M., Peebles, D., Heuer, H., Waddington, S.N., Raivich, G. (2010). Activation and deactivation of periventricular white matter phagocytes during postnatal mouse development. Glia. 58: 11-28.

Inserra, M.M., Bloch, D.A., and Terris, D.J. (1998). Functional indices for sciatic, peroneal, and posterior tibial nerve lesions in the mouse. Microsurgery 18,119-124.

Jungnickel, J., Haastert, K., Grzybek, M. Thau, N., Lipokatic-Takacs, E., Ratzka, A., Nölle, A., Claus, P., Grothe, C. (2010). Mice lacking basic fibroblast growth factor showed faster sensory recovery. Exp. Neurol. 223: 166-172.

Morgan, L., Jessen, K.R., and Mirsky, R. (1991). The effects of cAMP on differentiation of cultured Schwann cells: progression from an early phenotype (04+) to a myelin phenotype (P0+, GFAP-, N-CAM-, NGF-receptor-) depends on growth inhibition. J. Cell Biol**.** 112: 457-467.

Reichert, F., Saada, A. and Rotshenker, S. (1994). Peripheral nerve injury induces Schwann cells to express two macrophage phenotypes: phagocytosis and galagtose-specific lectin MAC-2. J. Neurosci. 14:3231-3245.

Seijffers, R., Mills, C.D., and Woolf, C.J. (2007). ATF3 increases the intrinsic growth state of DRG neurons to enhance peripheral nerve regeneration. J. Neurosci. 27 : 7911-7920.

Siconolfi, L.B., and Seeds, N.W. (2001). Mice lacking tPA, uPA, or plasminogen genes showed delayed functional recovery after sciatic nerve crush. J. Neurosci. 21 : 4348-4355.

Vogelaar, C.F., Vrinten, D.H., Hoekman, M.F., Brakkee, J.H., Burbach, J.P., Hamers F.P. (2004). Sciatic nerve regeneration in mice and rats: recovery of sensory innervation is followed by a slowly retreating neuropathic pain-like syndrome. Brain Res. 1027: 67-72.
